# Comparison of Deconvolution Filters for Photoacoustic Tomography

**DOI:** 10.1371/journal.pone.0152597

**Published:** 2016-03-31

**Authors:** Dominique Van de Sompel, Laura S. Sasportas, Jesse V. Jokerst, Sanjiv S. Gambhir

**Affiliations:** 1 Molecular Imaging Program at Stanford (MIPS), Stanford University School of Medicine, Stanford University, Stanford, CA 94305, United States of America; 2 Department of NanoEngineering, UC San Diego, La Jolla, CA 92093, United States of America; Shenzhen institutes of advanced technology, CHINA

## Abstract

In this work, we compare the merits of three temporal data deconvolution methods for use in the filtered backprojection algorithm for photoacoustic tomography (PAT). We evaluate the standard Fourier division technique, the Wiener deconvolution filter, and a Tikhonov L-2 norm regularized matrix inversion method. Our experiments were carried out on subjects of various appearances, namely a pencil lead, two man-made phantoms, an *in vivo* subcutaneous mouse tumor model, and a perfused and excised mouse brain. All subjects were scanned using an imaging system with a rotatable hemispherical bowl, into which 128 ultrasound transducer elements were embedded in a spiral pattern. We characterized the frequency response of each deconvolution method, compared the final image quality achieved by each deconvolution technique, and evaluated each method’s robustness to noise. The frequency response was quantified by measuring the accuracy with which each filter recovered the ideal flat frequency spectrum of an experimentally measured impulse response. Image quality under the various scenarios was quantified by computing noise versus resolution curves for a point source phantom, as well as the full width at half maximum (FWHM) and contrast-to-noise ratio (CNR) of selected image features such as dots and linear structures in additional imaging subjects. It was found that the Tikhonov filter yielded the most accurate balance of lower and higher frequency content (as measured by comparing the spectra of deconvolved impulse response signals to the ideal flat frequency spectrum), achieved a competitive image resolution and contrast-to-noise ratio, and yielded the greatest robustness to noise. While the Wiener filter achieved a similar image resolution, it tended to underrepresent the lower frequency content of the deconvolved signals, and hence of the reconstructed images after backprojection. In addition, its robustness to noise was poorer than that of the Tikhonov filter. The performance of the Fourier filter was found to be the poorest of all three methods, based on the reconstructed images’ lowest resolution (blurriest appearance), generally lowest contrast-to-noise ratio, and lowest robustness to noise. Overall, the Tikhonov filter was deemed to produce the most desirable image reconstructions.

## Introduction

Photoacoustic tomography (PAT) is a hybrid optical and ultrasound imaging technique that combines the high spatial resolution of ultrasound with the high optical contrast of diffuse optical imaging. It is a highly promising modality that has received increasing attention from the biomedical imaging community due to its ability to image in three dimensions, up to 5 cm deep into living tissue. PAT is based on the illumination of an object with brief pulses of laser light. As the light diffuses into the tissue, it is absorbed differentially by the various regions of the tissue. The light absorption causes brief localized heating and an increase in pressure proportional to the amount of light energy absorbed. This leads to the generation of pressure waves that travel out of the tissue and can be measured by ultrasonic transducers. The detected signals are then reconstructed into an image of optical absorption using a computer algorithm.

The field of photoacoustic tomography for biomedical imaging has seen an explosion of novel system geometries and reconstruction algorithms. Common system geometries include circular [1–3], semi-cylindrical or curved [3–5], linear [6–8], planar [9–13], hemispherical [14, 15] and spherical [16] measurement apertures, each with various numbers of ultrasound transducer elements. The system used in this study features a hemispherical bowl with 128 transducer elements (see Methods section). In parallel, an equally diverse range of reconstruction methodologies has been proposed. The main paradigms are one-step analytical backprojection algorithms [17–20], time reversal algorithms [21–25], and iterative model-based reconstruction methods [3, 26–32]. An overview of photoacoustic reconstruction methods and further references are given in [24], [33], [34] and [35]. Of these methods, time reversal and iterative methods generally offer superior image and system modeling capabilities. However, one-step analytical backprojection methods have been popular because of their speed and relative ease of implementation.

A key step in most backprojection algorithms is the deconvolution of the recorded pressure signals by the transducer impulse response, prior to backprojection into the reconstructed volume. The deconvolution operation is notoriously ill-posed, and differences in its implementation can have a large impact on the final quality of the reconstructed image, as demonstrated in this paper. The optimization of this operation is therefore of critical importance, and is the subject of this study. We compare the performance of three different deconvolution methods prior to filtered backprojection: division by the impulse response in the Fourier domain, the Wiener deconvolution filter, and a Tikhonov L-2 norm regularized matrix deconvolution scheme. These three techniques were chosen because they are widely known and used deconvolution methods that have proven successful in many signal processing applications. Since they are well-understood and easily implemented, we believe that the results of this study will be useful to any research groups looking to optimize the deconvolution step of PAT reconstruction algorithms. While division in the Fourier space [15, 36, 37] and Wiener filtering [5, 38] has been used in many previous studies, we believe that our study is the first to use Tikhonov L-2 norm regularized matrix deconvolution to deconvolve the pressure data in a filtered backprojection algorithm for photoacoustic tomography. To the best of our knowledge, this study is also the first to provide side-by-side comparisons of these three deconvolution techniques for 3D PAT.

Note that regularized matrix methods have been used previously in iterative reconstruction algorithms for PAT to achieve image smoothness or to implement compressed sensing. In those cases, however, regularization has been applied directly to the image domain, rather than the data domain. A notable exception was the data restoration technique proposed by Wang et al. [39], which used an L-1 norm penalty to sparsify a curvelet representation of the sinogram in 2D PAT. However, this method differs considerably from the signal-by-signal Tikhonov L-2 norm regularized matrix deconvolution explored in this paper. Wang et al.’s method is also less general in that it assumes that the pressure data can be arranged in a data space where small features trace out smooth sinusoidal paths. This may not be true for sparse sampling geometries.

The remainder of this paper is structured as follows. The Methods section explains the photoacoustic signal model, the three deconvolution methods, filtered backprojection, the scanner geometry, and the imaging subjects used. We also explain the performance metrics used to quantify image quality, and mention that we monitored the water temperature to obtain accurate values for the speed of sound. The Results section optimizes the parameters of the Wiener and Tikhonov filters, assesses the image quality afforded by each of the three deconvolution methods, and examines the robustness of each method to changes in noise levels. All photoacoustic data was acquired on the Nexus 128 scanner manufactured by Endra Life Sciences (Ann Arbor, MI, USA). Lastly, the Conclusions section summarizes the findings of our work, and the Further Work section gives recommendations for future work.

## Methods

### Photoacoustic waves and recorded pressure signal model

Photoacoustic pressure wave propagation can be described by the general wave equation

$$\begin{array}{c} \left(\nabla^{2}-\frac{1}{v_{s}^{2}}\frac{\partial^{2}}{\partial t^{2}}\right)p\left(r\prime ,t\right)=-\frac{\beta }{C_{p}}\frac{\partial }{\partial t}H\left(r\prime ,t\right). \end{array}$$ (1)

where *r*′ represents the coordinates anywhere in 3D space, *t* is time, *C*_*p*_ is the specific heat at constant pressure, *v*_*s*_ is the local speed of sound, *β* is the isobaric volume expansion coefficient, and *H* is the heat source function. Using this model, and assuming that *v*_*s*_ is constant everywhere, Wang et al. [37] showed that the pressure signal *p*_*d*_(*r*, *t*) recorded by a point transducer located at a particular position *r* is given by

$$\begin{array}{c} p_{d}\left(r,t\right)=\frac{\beta r_{p}}{4\pi C_{p}k}\left(\frac{1}{t}\int \int_{|r-r\prime |=v_{s}t}A\left(r\prime \right)dS\right)*p_{d0}\left(t\right) \end{array}$$ (2)

where *A*(*r*′) is the absorbed optical energy distribution, *r*_*p*_ is the distance between the transducer and the point source, and *p*_*d*0_(*t*) is the pressure recorded by the transducer after illuminating a point source, with a delay time of −*r*_*p*_ *v*_*s*_. As described by Wang et al. [37], *p*_*d*0_(*t*) = *p*_0_(*t*)*h_*t*_(*t*), where *p*_0_(*t*) is the true pressure signal from the point source arriving at the transducer (again with a delay time of −*r*_*p*_ *v*_*s*_), and *h*_*t*_(*t*) is the impulse response of the transducer. The constant *k* is defined by $k=\frac{p_{0}\left(t\right)r_{p}}{I\prime \left(t\right)}$, where *I*(*t*) is the temporal illumination function, and $I\prime \left(t\right)=\frac{d}{dt}I\left(t\right)$. From here on, we refer to the signals *h*_*t*_(*t*) and *p*_*d*0_(*t*) = *p*_0_(*t*)*h_*t*_(*t*) as the transducer impulse response and the *overall* scanner impulse response, respectively. Finally, note that point detectors are an idealization not achievable in practice. However, the assumption of point detectors is commonly made in the literature on photoacoustic tomography and has been used with great success, including in the paper by [37].

### Deconvolution methods

The absorbed optical energy distribution *A*(*r*′) can be reconstructed in two steps. First, we recover the spherical projections by deconvolving Eq 2. Note that these deconvolutions represent *temporal* deconvolutions, i.e. deconvolutions of the recorded pressure signals. Second, we backproject the projections using a filtered backprojection (FBP) method. In this section, we discuss the three deconvolution schemes compared in this paper. The FBP method is explained in the next section.

#### Fourier division deconvolution

The simplest method for deconvolving Eq 2 is to divide out the overall scanner impulse response *p*_*d*0_(*t*) in the Fourier domain [37]. This operation can be written as

$$\begin{array}{c} \int \int_{|r-r\prime |=v_{s}t}A\left(r\prime \right)dS=\frac{4\pi C_{P}kt}{\beta r_{p}}F^{-1}\left[\frac{F[p_{d}\left(r,t\right)]W\left(\omega \right)}{F[p_{d0}\left(t\right)]}\right] \end{array}$$ (3)

The operators ℱ and ℱ^−1^ represent the Fourier transform and inverse Fourier transform, respectively. Note that these represent Fourier transforms of temporal signals (recorded pressure signals). The window function *W*(*ω*) band-limits the recorded pressure signals in order to prevent unwanted high frequency amplification by the division by ℱ[*p*_*d*0_(*t*)]. We used a standard sine window, given by $W\left(\omega \right)=|\sin(\pi \bar{\omega }/2)|$, where $\bar{\omega }=\frac{\omega }{\omega_{max}}\in \left[-1,1\right]$.

#### Wiener deconvolution

The window function *W*(*ω*) is only a heuristic method for countering the sensitivity of Fourier division to high frequency noise. Wiener deconvolution represents a more rigorous approach for improving robustness to noise. It explicitly models the power densities of the noise and deconvolved signal, and solves for a deconvolved signal that minimizes the expected mean square error [40]. To simplify our notation, let us rewrite the signal model as

$$\begin{array}{c} y\left(t\right)=x\left(t\right)*h\prime \left(t\right)+v\left(t\right) \end{array}$$ (4)

where *y*(*t*) is the observed signal, *x*(*t*) is the unknown input signal, * denotes convolution, *h*′(*t*) is the impulse response function, and *v*(*t*) is some unknown additive noise, independent of *x*(*t*). In our case, *y*(*t*) = *p*_*d*_(*r*, *t*), *h*′(*t*) = *p*_*d*0_(*t*), and $x\left(t\right)=\frac{\beta r_{p}}{4\pi C_{P}k}(\frac{1}{t}\int \int_{|r-r\prime |=v_{s}t}A\left(r\prime \right)dS)$. The Wiener deconvolution method computes the input signal estimator $\hat{x}$ given by

$$\begin{array}{c} \hat{X}\left(f\right)=Y\left(f\right)G\left(f\right), \end{array}$$ (5)

where $\hat{X}\left(f\right)$ and *Y*(*f*) are the Fourier transforms of the input signal estimator $\hat{x}\left(t\right)$ and the observed signal *y*(*t*), respectively, and the filter *G*(*f*) is given by

$$\begin{array}{c} G\left(f\right)=\frac{H^{*}\left(f\right)S\left(f\right)}{{\left|H\left(f\right)\right|}^{2}S\left(f\right)+N\left(f\right)}. \end{array}$$ (6)

The superscript * in Eq 6 denotes complex conjugation, |.| denotes the magnitude of a complex number, and *H*(*f*) is the Fourier transform of *h*′(*t*). Next, *S*(*f*) is a model for the mean power spectral density (PSD) of the input signal *x*(*t*), and *N*(*f*) is the mean power spectral density of the noise *v*(*t*). The estimator $\hat{x}\left(t\right)$ is recovered by computing the inverse Fourier transform of $\hat{X}\left(t\right)$. We assume that the scanner impulse response *h*′(*t*) = *p*_*d*0_(*t*) is the same for all transducers. Next, we estimate *N*(*f*) from the average frequency spectrum of the leading parts of the pressure signals, corresponding to time points before any photoacoustic waves can travel from the imaged object to the transducer elements.

Lastly, we assume a Gaussian profile for *S*(*f*), and set the area under the curve of *S*(*f*) = *G*(*f*;*μ*, *σ*) over the frequency range *f* = 0 MHz to *f*_*Nyquist*_ = 10 MHz to be equal to the corresponding area under the power spectral density of a standard Fourier deconvolution given by *Y*(*f*)/*H*(*f*), where *Y*(*f*) and *H*(*f*) are again the Fourier transforms of the measured signal and the scanner impulse response *h*′(*t*) = *p*_*d*0_(*t*), respectively. We let *μ* = 0. The value of *σ* that optimizes the reconstructed image quality is determined in the Results section. The rationale for using a Gaussian PSD is based on the well-known fact that most of the spectral power in natural images is concentrated towards the lower spatial frequencies. More precisely, natural images’ power spectra fall as the spatial frequency increases, and are relatively isotropic [41]. In this paper, we decided to apply the Gaussian model to the representation of the PSD of the deconvolved signals, which are essentially spherical projections of the reconstructed images, and hence should have frequency contents similar to the frequency content of the original 3D image. To support this last claim, consider the following argument. If the projections were planar and parallel, the 1D Fourier transform of the projections would sample 1D lines through the original image’s 3D Fourier space perpendicular to the projection planes, as described by the Fourier Slice Theorem for the classical Radon transform [42]. Since the reconstructed image volume is small compared to the radius of the imaging bowl, we expect the spherical surfaces to be near-parallel within the confines of the reconstruction volume, and the predictions by the Fourier Slice Theorem to be approximately valid (this approximation is also known as the ‘far field approximation’ [42]). In other words, if the PSD of the 3D Fourier representation of the imaging subject can be modeled by a Gaussian distribution, 1D lines through that space will be adequately modeled by Gaussian distributions as well, which in turn justifies our choice of a Gaussian profile for the deconvolved signals’ mean power spectral density *S*(*f*).

#### Tikhonov L-2 norm deconvolution

A powerful alternative to Fourier-formulated deconvolution methods is given by matrix deconvolution methods. Continuing the notation used above, we represent the measured signal *y*(*t*) by a column vector *y*, and the unknown input signal *x*(*t*) by a column vector *x*. We then declare a non-circular convolution matrix *C* that contains increasingly shifted copies of the scanner impulse response *p*_*d*0_(*t*) along its columns. Hence, for each signal, the deconvolution problem takes the form

$$\begin{array}{c} y=Cx, \end{array}$$ (7)

which can be solved for the input signal *x*. To regularize the matrix inversion, we use the standard Tikhonov technique [43], minimizing a cost function of the form

$$\begin{array}{c} \phi \left(x\right)=||Cx-{y||}^{2}+{\left||\Gamma x|\right|}^{2} \end{array}$$ (8)

where Γ is a matrix that determines the nature of the regularization. We choose to minimize the L-2 norm of the solution, primarily because it is the most convenient form of regularization. This is done by setting Γ = *βI*, where *β* is a parameter setting the strength of the regularization, and *I* is the identity matrix. The value of *β* that optimizes the reconstructed image quality is determined in the Results section. Lastly, note that other forms of regularization such as L-1 norm regularization are possible as well, and will be investigated in future work (see Further Work section). The solution to Eq 8 is given by

$$\begin{array}{c} \hat{x}={\left(C^{T}C+\Gamma^{T}\Gamma \right)}^{-1}C^{T}y \end{array}$$ (9)

where $\hat{x}$ represents the regularized estimate of the deconvolved pressure data. In this work, the size of the non-circular convolution matrix *C* was 512 × 512. Its rank was 502, confirming the ill-posedness of the inversion problem. By comparison, the rank of the circular implementation of *C* (also of size 512 × 512) was 512. Results for both circular and non-circular implementations of *C* are shown in the Image domain section of the Results section. As explained above, the ill-posedness problem was addressed using the Tikhonov L2 norm regularization. Finally, Eq 9 was solved analytically using the backslash operator in Matlab.

### Filtered backprojection

**Fig 1:**
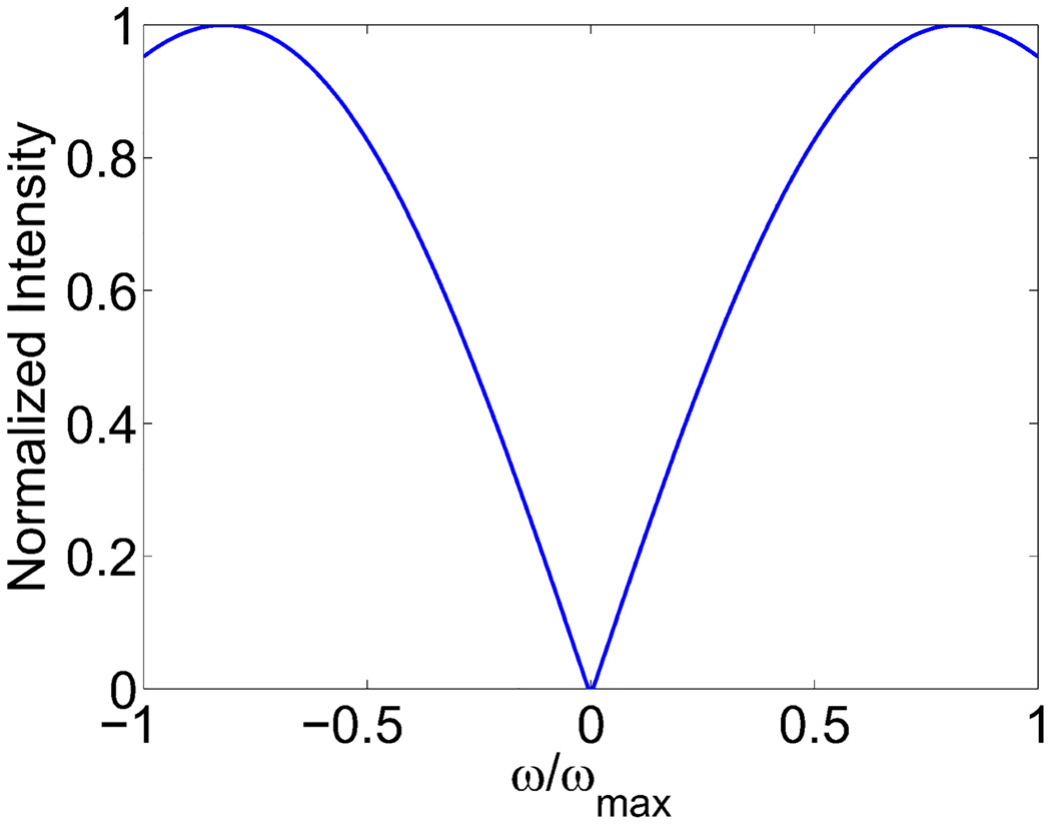
Sinc filter for filtered backprojection algorithm.

After deconvolving the pressure signals, the 3D photoacoustic image can be reconstructed using a filtered backprojection algorithm. As the name suggests, this method first filters the data and then backprojects it into the reconstruction volume. To prevent excessive noise amplification, it is common to use a filter with a high-frequency roll-off, rather than the standard ramp filter. We used the common sinc filter, given by

$$\begin{array}{c} R\left(\bar{\omega }\right)=\left|\frac{1}{a}\sin\left(a\pi \bar{\omega }\right)\right|{\left(\frac{\sin\left(a\pi \bar{\omega }\right)}{a\pi \bar{\omega }}\right)}^{2} \end{array}$$ (10)

where $\bar{\omega }=\frac{\omega }{\omega_{max}}\in \left[-1,1\right]$, and *ω* is the spatial frequency of the signal to be backprojected. By changing the value of *a*, one can tune the filter to obtain various degrees of high frequency roll-off. We empirically found that a value of *a* = 0.38 gave desirable results, based on a visual appreciation of the images. The filter for this value is shown in Fig 1. Further optimization of this value is deferred to future work. Here we focus on the comparison between the three deconvolution methods while keeping the sinc filter constant. The backprojection operation was implemented on a GeForce GTX 590 GPU using the Accelereyes Jacket package in Matlab, reconstructing a 200 × 200 × 200 volume from 120 views in approximately 60 seconds.

### Photoacoustic scanner

**Fig 2:**
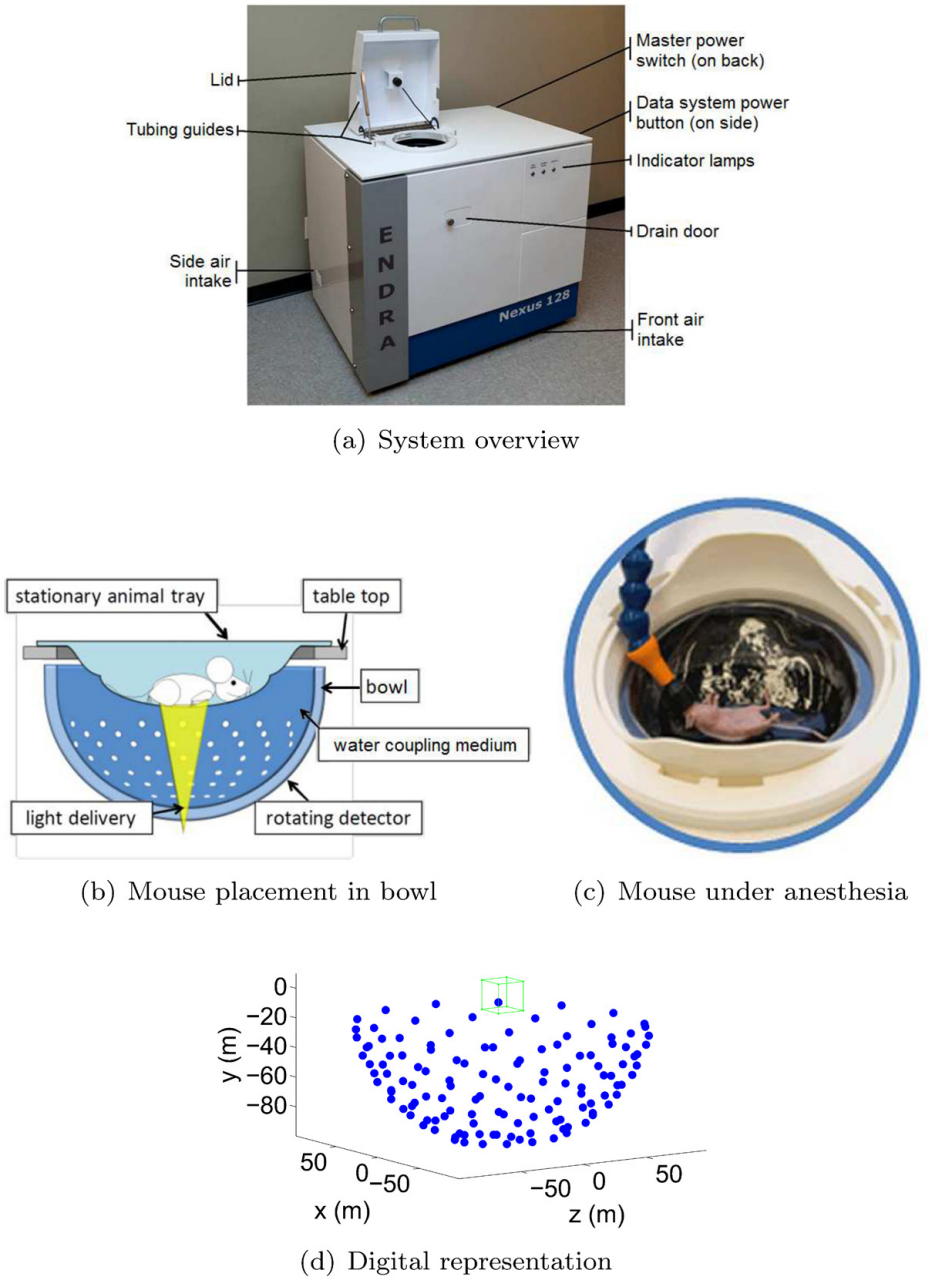
Nexus 128 scanner. (a-c) are reproduced with permission from http://www.endrainc.com. The green box in (d) delineates the spatial support of a representative reconstructed field of view. The dimensions of this reconstructed volume are typically set to 2 × 2 × 2 cm, and the isotropic reconstruction resolution typically to 0.2 mm. The blue dots show the locations of the 128 transducers in the bowl.

**Fig 3:**
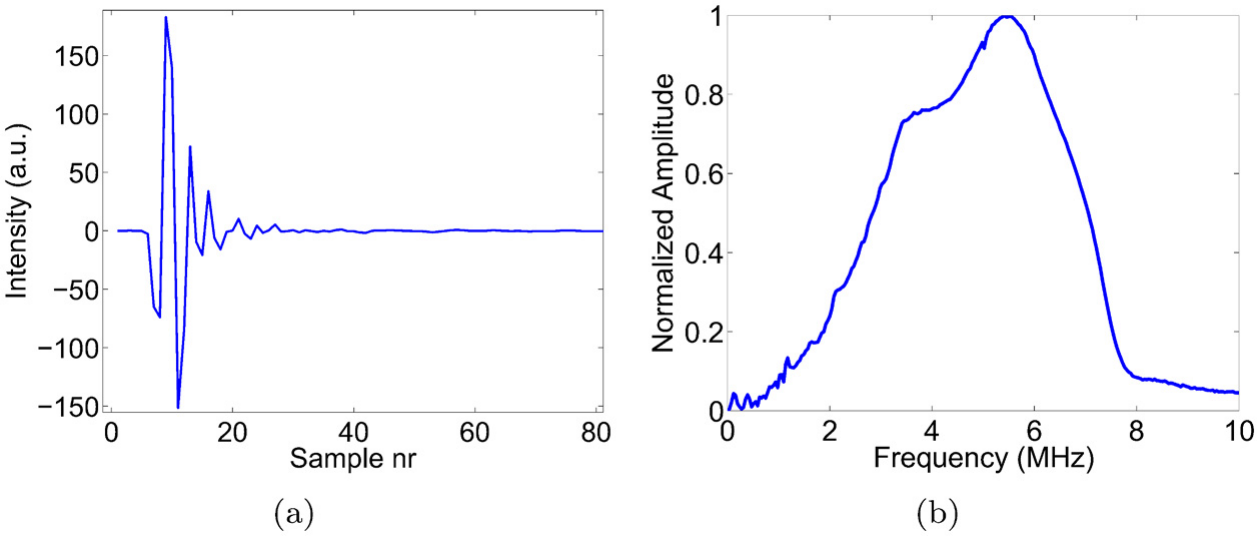
(a) Overall scanner impulse response *p*_*d*0_(*t*) = *p*_0_(*t*)*h_*t*_(*t*), and (b) its frequency spectrum.

The photoacoustic scans for this study were acquired using the Nexus 128 scanner, manufactured by Endra Life Sciences (Ann Arbor, MI, USA). Fig 2 illustrates the system. The detection geometry consists of 128 unfocused transducers arranged on a hemispherical bowl. The radius of the bowl is 101.3 mm. A plastic tray is placed on top of the bowl for animal positioning. The tray is covered by a lid that must be closed while scanning (an interlock prevents scanning while the lid is open) as a protective measure to rule out the possibility of eye damage from the laser to the user. A webcam inside the lid allows users to monitor the mouse during a scan. The bowl is filled with water to provide acoustic coupling. A water heating system maintains the water temperature at 38°C between scans to enable small animal imaging. The readings of the water heating system’s thermometer can be accessed in a log file. The water heating pump is switched off during scans to eliminate the risk of bubble formation. The blue dots in Fig 2(d) indicate the transducer positions, and the green box indicates the spatial support of a standard reconstructed volume (200 × 200 × 200 with an isotropic resolution of 0.1 mm centered on the focal center of the hemispherical bowl). The maximum field of view supported by the system is currently a sphere of radius 3.8 cm, again centered on the focal center of the hemispherical bowl. Each transducer has a circular detection surface with a diameter of 3 mm and records at a sampling rate of 20 MHz. The center frequency of the transducers is 5 MHz with a bandwidth of approximately 70%. Note that if the bandwidth were lower, the transducers would detect fewer sound frequencies in the lower and higher ranges. In the image space, this would translate into fewer low and high spatial frequencies, i.e. lower contrast for larger masses and blurrier edges for small details, respectively. The scanner impulse response *p*_*d*0_(*t*) was measured by the scanner’s manufacturer by imaging a hair-like mylar thread, approximately 75 *μm* in diameter, which was placed directly in the water at the isocenter of the detector bowl. The signal *p*_*d*0_(*t*) was measured without the standard diverging lens of the scanner to obtain a higher than normal laser power (43 mJ per pulse) and hence to ensure a high signal-to-noise measurement. The scanner impulse response *p*_*d*0_(*t*) was assumed equal for all transducers, and is shown in Fig 3, along with its frequency content. To increase geometric sampling of the detection surface, the bowl is rotated in steps through multiple positions during a single scan. Usually, approximately 120 to 180 bowl positions, distributed uniformly over 360 degrees, suffice to obtain a high quality reconstructed image. Each such bowl position will be referred to as a ‘view’ in the remainder of this paper. Due to the small angular steps and light weight of the bowl, no significant shaking of the transducers nor bubble formation was observed as the stepper motor rotated the bowl.

The scanner uses a tunable, near-infrared Nd:YAG laser that produces 7 ns pulses at up to 25 mJ per pulse. The typical laser power during our scans was 5 mJ per pulse. The wavelength can be tuned within a wavelength range of 680–980 nm. The repetition rate of the laser is 20 Hz. To reduce the effect of laser pulse power variations, and to increase the SNR of the recorded pressure signals, we average signals over a predetermined number of pulses (typically 100). At the laser repetition rate of 20 Hz, a standard scan using 180 bowl positions therefore takes 15 minutes per excitation wavelength. Note that the scanner allows faster frame rates and hence lower total scan times by using fewer laser pulses per bowl position. However, this would come at the expense of reduced signal averaging to lower signal to noise ratios, and hence poorer final image qualities.

### Dependence of speed of sound on water temperature

The speed of sound was corrected for changes in the water temperature during each scan. The importance of doing so was demonstrated by [44]. As mentioned, the water heating system of the scanner is switched off while scanning to minimize the risk of air bubble formation. This causes the water temperature to drop gradually during each scan, on the order of 1.5°C during a 15 minute, 180 view scan. To compensate for this effect, we continuously measured and recorded the temperature of the water coupling medium. To deduce the speed of sound from these temperature measurements, we used the nine-term Mackenzie equation [45], given by

$$\begin{array}{c} v=a+bT+cT^{2}+dT^{3}+e\left(S+f\right)+gD+hD^{2}+iT\left(S+f\right)+jTD^{3} \end{array}$$ (11)

where *v* is the speed of sound in meters per second, *T* is the water temperature in degrees Celsius, *S* is the salinity in parts per thousand and *D* is the depth in meters. The coefficients of Eq 11 can be found in [45] or [44]. The salinity *S* and depth *D* were both set to 0.

### Imaging subjects

**Fig 4:**
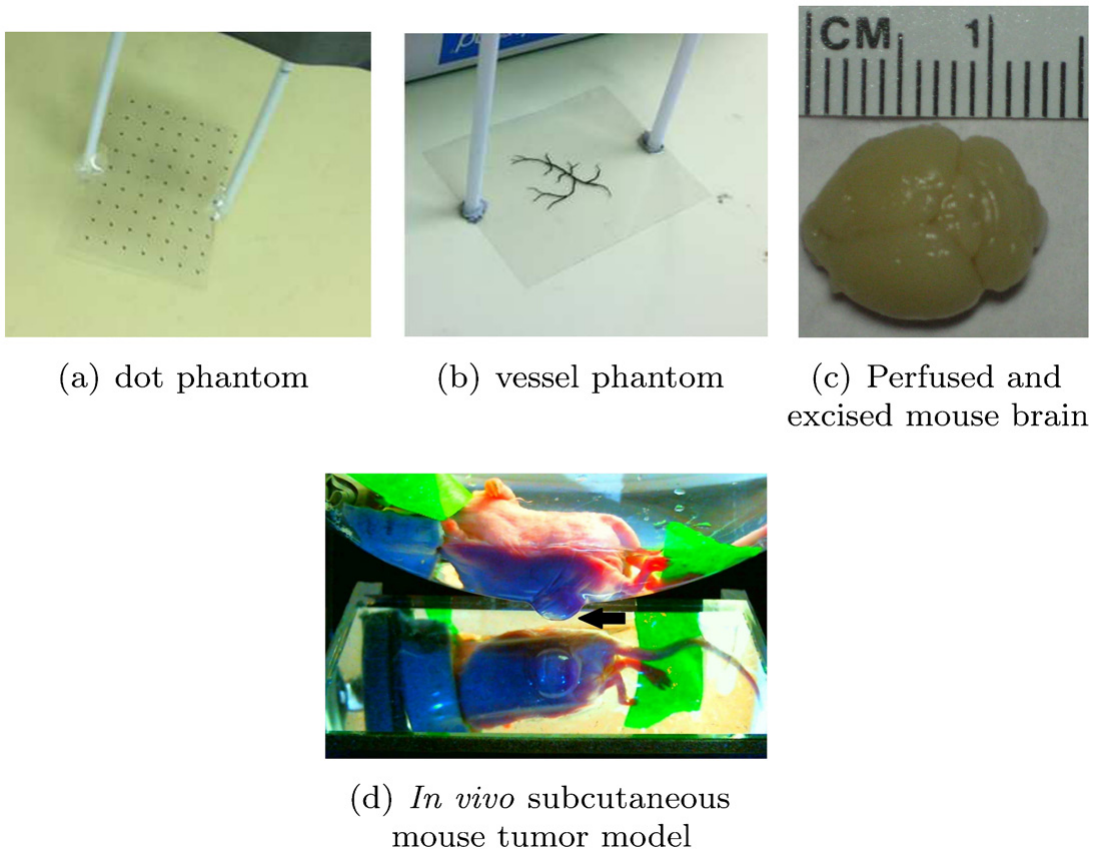
Imaging subjects. (a, b) Dot and vessel phantoms printed onto transparent overhead projector film using a standard black and white laser printer. (c) Perfused and excised mouse brain. Left side: front of the brain. Right side: rear of the brain (cerebellum). (d) *In vivo* subcutaneous mouse tumor model. Notice the dimple at the bottom of the plastic tray (indicated by the black arrow), which contains a small amount of coupling fluid (water), and is used to help position the subcutaneous tumor. The diameter of the dimple is 15 mm.

In this study, the imaging subjects used were an approximately spherical pencil lead point (0.3 mm in diameter), two physical phantoms, an *in vivo* subcutaneous mouse tumor model and a perfused and excised mouse brain. All animal work was conducted in accordance with the guidelines of and approved by the Administrative Panel on Laboratory Animal Care at Stanford University. Tumors were allowed to grow up to 1000 mm^3^. Animals were euthanized if they showed hunched posture, unkempt coat, or change in body weight more than 20% of baseline. No animals died during any point of the scanning procedures. Animals were given access to standard water and diet ad libitum and housed no more than 5 per cage. Animals were housed in a negative pressure vivarium with standard 12 hour light/dark cycles. The phantoms were produced by printing a digital design onto a transparent overhead projector film using a standard black and white laser printer (Xerox Phaser 3600 PS). The print resolution was 600 × 600 dpi, and the ink used was from a standard Xerox Phaser 3600 PS black and white toner cartridge. The thickness and material of the transparency were 0.1 mm and cellulose acetate, respectively. The designs are shown in Fig 4(a) and 4(b), where the white straws used to lower the phantoms into the water are also shown. The dots were 0.6 mm in diameter. The maximum width of the vessel phantom was 0.4 mm. The digital design of the vessel phantom was obtained from the k-Wave Toolbox for Matlab [46].

The *in vivo* subcutaneous mouse tumor model was generated as follows. MDA-435S cells were acquired from ATCC and grown in DMEM and passaged when they reached 80% confluence. Female nu/nu mice age 6–16 weeks were used. Prior to imaging, the mice were anesthetized with 2% isofluorane in house oxygen at 2 L/min and confirmed with tail pinch. For xenograft tumors, we created a cell pellet containing 106 cells in 50% growth factor reduced matrigel (Becton Dickinson) in media and implanted the pellet subcutaneously on the hind limb of a nude mouse. The tumors were imaged when they reached approximately 10 mm in diameter. A picture of the mouse positioned in the plastic tray of the scanner is shown in Fig 4(d). Note that the tray contains a dimple to immobilize the tumor, and to position it in the center of the laser beam. As seen in the Results section, the tumor used in this study was selected for its particularly tortuous and pervasive vasculature.

**Fig 5:**
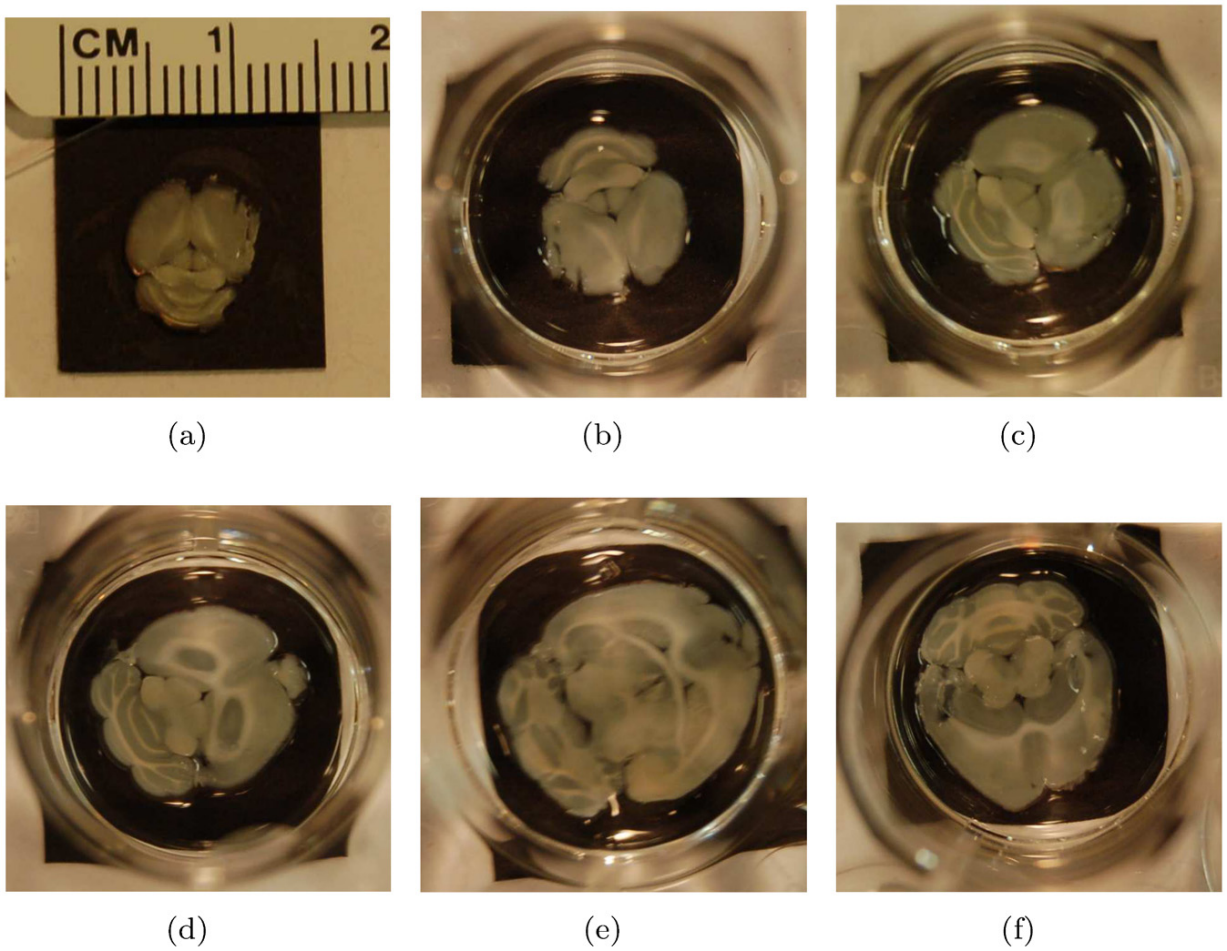
Representative slices through the perfused and excised mouse brain.

Lastly, the mouse brain perfusion and excision was performed as follows. After placing the mouse under deep anesthesia, the animal’s chest and abdomen were exposed by making a surgical cut along the midline. The frontal rib cage was cut, exposing the pericardium. A large gauge needle (18 gauge) connected to a syringe by a long PE tube was inserted into the left ventricle of the heart. The inferior vena cava was severed with scissors to allow the blood, saline and fixative to leave the body during perfusion. Using a syringe pump, the mouse was perfused with 20 mL of saline for 5–10 min followed by 20 mL of 10% formalin fixative solution. After perfusion, a craniotomy was performed and the brain was excised carefully and stored in 10% formalin. The perfused and excised mouse brain is shown in Fig 4(c). A collection of representative slices through the mouse brain, obtained after the photoacoustic scan, is shown in Fig 5.

### Simulation of noise in scan data

To assess each deconvolution method’s robustness to noise, we simulated noise onto high quality scan data. This data was obtained by scanning with a high number of averaging pulses (so as to achieve a low level of noise in the recorded pressure signals) and a geometric sampling density high enough to eliminate streak artifacts. These requirements were met by choosing 100 averaging pulses and 180 bowl positions. Higher levels of noise were then simulated by adding zero-mean Gaussian noise to the recorded pressured data. Gaussian noise was chosen because it adequately describes the noise variations that were observed around averaged signals in experimental measurements. We decided to simulate noise, rather than experimentally acquire repeated scans with successively lower pulse numbers, to ensure consistency and comparability between the reconstruction results. By simulating, we prevented confounding effects such as motion artifacts, laser power drift and inter-scan changes in water temperature (i.e. speed of sound) from biasing our results.

### Deconvolution filter parameter optimization

The Wiener and Tikhonov methods require the setting of tunable parameters, namely *σ* and *β*. To optimize these values, one could simply use trial and error based on a visual assessment of the reconstructed images. In this work, however, we use a more systematic approach by assessing the effect of changing parameter values in both the pressure data (projection) and image domains.

For the pressure data domain, we assess the ability of each filter to recover the ideal flat frequency spectrum of a deconvolved impulse signal as a function of the respective parameter value. Our metric for this assessment is the normalized correlation coefficient (NCC) between the ideal flat spectrum and the actual recovered spectrum, given by

$$\begin{array}{c} C=\frac{<S(f),I(f)>}{\left||S(f)||.||I(f)|\right|} \end{array}$$ (12)

where *S*(*f*) is the spectrum of the deconvolved impulse signal, *f* denotes frequency, *I*(*f*) is the ideal flat spectrum, and <.,.> denotes the dot product. The spectra are treated as column vectors, whose norm is denoted by ||.||. In this experiment, impulse signals were measured by scanning a piece of pencil lead measuring 0.3 mm in diameter. Since we used 128 bowl positions with 128 transducer positions each, the scan yielded 16,384 signals. To arrive at a single spectrum *S*(*f*) for any parameter value, we computed the mean spectrum of all 16,384 deconvolved signals. The value of *C* ranges between −1 and 1; a perfect correlation is indicated by 1. Hence, we regard the optimal filter setting as the one that corresponds to the highest value of *C* (i.e. closest to 1). The results of this optimization experiment are presented in the Projection domain section of the Results section.

For the image domain, we compute noise versus resolution trade-off curves, as generated by varying the filters’ parameter values. This is achieved by plotting the full width at half maximum (FWHM) of a 1D Gaussian fit to the reconstructed pencil point versus the sum of squared differences between neighboring pixels in a background patch, normalized by the sum of the squares of the intensities in that patch. FWHM is a common metric for quantifying image resolution. The sum of squared differences between neighboring pixels is commonly used in the computed tomography literature as a metric for describing image noise, where it is usually exploited to regularize against such noise (i.e. to reduce it) [47]. The results of the noise versus resolution trade-off analysis are shown in the Image domain section of the Results section. We also combine the results from the Projection domain and Image domain sections, and identify the optimal parameter values.

### Image quality metrics

**Fig 6:**
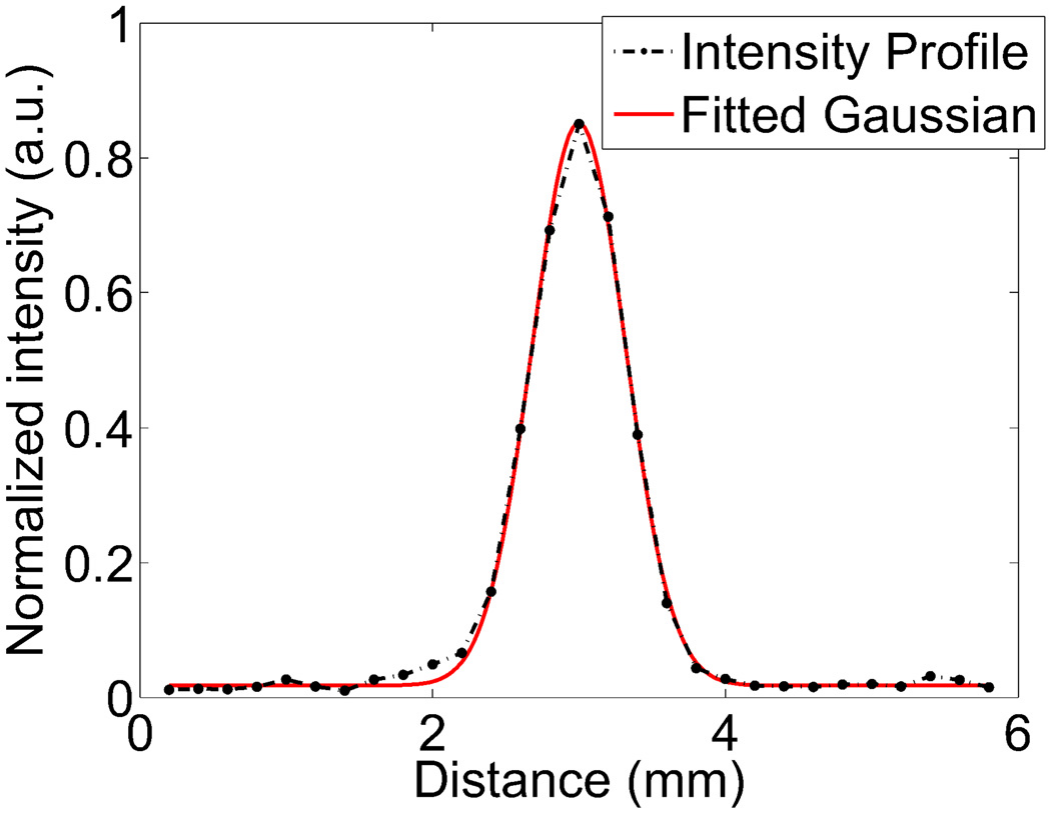
Example Gaussian fit to a 1D intensity profile in order to estimate the FWHM and CNR of point-like or linear features.

After tuning the parameters of the deconvolution filters, we reconstructed each of the remaining imaging subjects presented in the Imaging Subjects section using each deconvolution method, and compared the resulting image qualities both visually and quantitatively. The quantitative metrics used were the full width at half maximum (FWHM), as well as the contrast-to-noise ratio (CNR) of selected point and linear image features. Similar to before, the FWHM and CNR were computed by fitting Gaussian functions to 1D intensity profiles through selected point and line features. The FWHM was given by the width of the Gaussian at half maximum, and the CNR was given by

$$\begin{array}{c} \text{CNR}=\frac{A}{\sigma_{b}} \end{array}$$ (13)

where *A* is the amplitude of the fitted Gaussian, and *σ*_*b*_ is the standard deviation of the intensities in a selected background region. For the line profiles, of which several examples are shown in the Results section, this background region was chosen to be the part of the line profile more than three standard deviations away from the mean of the fitted Gaussian. An example Gaussian fit is shown in Fig 6. For the mouse brain reconstruction shown in the Results section, the examined linear feature was not as spatially isolated as the dots and linear features in the other imaging subjects. Hence we estimated the CNR of this feature by dividing its average peak-to-trough difference by the standard deviation of the area outside of the brain slice.

For the robustness to noise experiments, we used a global CNR metric, computed over the entire image. This was done because the Gaussian fitting to 1D line profiles became unstable at high noise levels. The metric used was the overall contrast-to-noise ratio (CNR_*o*_), defined by

$$\begin{array}{c} CNR_{o}=\frac{\bar{F}-\bar{B}}{\sigma_{B}} \end{array}$$ (14)

where

$$\begin{array}{c} \bar{F}=\frac{\sum_{j=1}^{N}x_{j}R_{j}}{\sum_{j=1}^{N}R_{j}} \end{array}$$ (15)

and where *x*_*j*_ is the *j*^*th*^ voxel of the reconstructed image, containing a total of *N* voxels, and where *R*_*j*_ is the *j*^*th*^ voxel of a normalized reference image. In this work, the normalized reference image *R* is the highest quality reconstruction of each object (i.e. with no added noise), whose intensities were scaled to the 0 to 1 range. In other words, *R* acts as a soft mask for the overall CNR computation. The variable $\bar{F}$, then, represents a weighted average of the reconstructed image, estimating the average of the images’ foreground. To compute the average background value, we similarly define

$$\begin{array}{c} \bar{B}=\frac{\sum_{j=1}^{N}x_{j}\left(1-R_{j}\right)}{\sum_{j=1}^{N}\left(1-R_{j}\right)} \end{array}$$ (16)

where the weights (1 − *R*_*j*_) weight in favor of the background regions. Lastly, we use the weighted standard deviation

$$\begin{array}{c} \sigma_{B}=\sqrt{\frac{\sum_{j=1}^{N}\left(1-R_{j}\right){\left(x_{j}-\bar{B}\right)}^{2}}{\sum_{j=1}^{N}\left(1-R_{j}\right)}} \end{array}$$ (17)

Note that Eqs 15, 16 and 17 are simply weighted extensions of the standard definitions for the mean ($\bar{x}=\frac{1}{N}\sum_{j=1}^{N}x_{j}$) and standard deviation ($\sigma =\sqrt{\frac{1}{N}\sum_{j=1}^{N}{\left(x_{j}-\bar{x}\right)}^{2}}$). For purely binary (’hard’) masks *R*, where the intensity values are either 0 or 1, the definitions revert to their standard forms [48]. Lastly, for the sake of consistency, we used the same mask for all reconstructions of a particular object, regardless of noise level or deconvolution method. This mask was obtained for each subject by computing the mean of the normalized reference images obtained by using each of the deconvolution methods.

## Results

In the following sections, we optimize the Wiener and Tikhonov filters’ parameters and compare the performance of all three deconvolution methods. At this point, we note that the computational expense was comparable for each of the three deconvolution methods. The times to deconvolve pressure data from 180 views were 1.6, 3.2 and 1.9 seconds for the Fourier division, Wiener and Tikhonov approaches, respectively. The longer computation time for the Wiener filter stems from the fact that it uses a preliminary Fourier division deconvolution to estimate the total power in the signal power density function *S*(*f*).

### Filter parameter optimization

In this section, we characterize the performance of the deconvolution filters under changing parameter values in both the projection domain and the image domain. In the former, we characterize the frequency response of the deconvolution of recorded ultrasound data; in the latter, we perform an analysis of noise versus resolution in the image domain after backprojection.

#### Projection domain

##### Fourier filter

**Fig 7:**
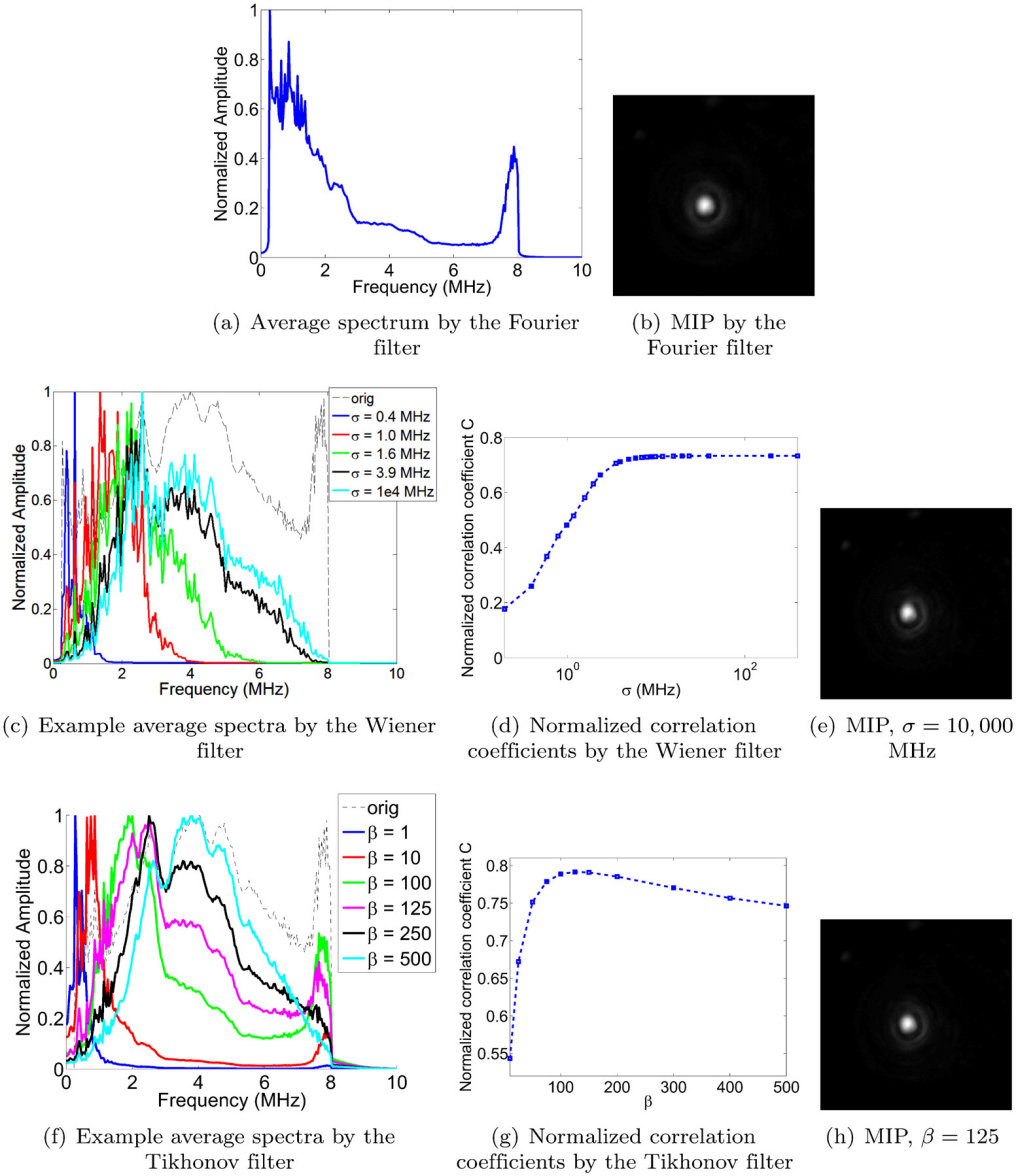
Frequency spectra of deconvolved pencil point signals, filter parameter optimization, and maximum intensity projections (MIP). (a-b) Average Fourier spectrum of the deconvolved pencil point signals, and MIP, using Fourier filter. The normalized correlation coefficient (NCC) *C* is equal to 0.6620. (c-e) Wiener filter example spectra and normalized correlation coefficients. ‘Orig’ stands for the average frequency spectrum before deconvolution. The maximum NCC value was achieved as *σ* tended to infinity (uniform distribution), with *C* tending to 0.733. Note that convergence was already reached for values of *σ* ≈ 20MHz and above. (f-h) Tikhonov filter example spectra and normalized correlation coefficients. Maximum NCC achieved at *β* = 125 and *C* = 0.7914. The image intensities of the reconstructions are normalized (black: 0, white: 1), and the dimensions of the MIP images are 10 × 10 mm.

For the sake of comparison with the Wiener and Tikhonov filters, we began by examining the Fourier filter’s performance when deconvolving pressure signals from a pencil point source. Note though that our Fourier filter design in Eq 3 did not contain any scalable parameters. Fig 7(a) and 7(b) shows the average frequency spectrum of the Fourier deconvolved pencil point signals, as well as a maximum intensity projection of the reconstructed pencil lead. The normalized correlation coefficient obtained for the Fourier deconvolution filter was *C* = 0.6620.

##### Wiener: optimization of the parameter *σ*

Fig 7(c)–7(e) show the results of the normalized correlation coefficient analysis for the Wiener filter, namely example frequency spectra, the normalized correlation coefficient (NCC) curve as a function of *σ* (where *σ* is as defined in the Methods section), and a maximum intensity projection of the pencil lead reconstruction at *σ* = 10,000 MHz. In Fig 7(c), we observe that the curves expand from left to right as *σ* increases, and that the curves converge around *σ* = 20MHz and above. The values of *σ* in Fig 7(c) were chosen to illustrate this trend with roughly equally spaced curves. All curves for *σ* = 20MHz or above converged onto the cyan curve shown in the plot. As shown in Fig 7(d), the NCC curve converges to an optimal value of C = 0.733 for *σ*’s greater than ∼20 MHz. We decided to use a value of 10,000 MHz in all of our further reconstructions, but note that we could have also used any other value above the convergence point of the NCC curve, such as 100MHz as 1,000 MHz; the image quality was insensitive to the value of *σ* as soon as it surpassed 20MHz. Figure A in S1 File (supporting information to this paper) confirms that this optimal value holds for the other imaging subjects as well, by showing image reconstructions as a function of *σ*. Note that as *σ* tends to infinity, *S*(*f*) tends towards a uniform distribution. As explained in the Methods section, the area under the Gaussian curve between the frequency range *f* = 0 MHz and *f*_*Nyquist*_ = 10 MHz is scaled to one before further scaling by the total expected power. This prevents the area under the Gaussian curve between *f* = 0 MHz and *f*_*Nyquist*_ = 10 MHz from tending to zero as *σ* tends to infinity.

##### Tikhonov: optimization of the parameter *β*

Mirroring the section above, Fig 7(f)–7(h) show example spectra as well as the resulting normalized correlation coefficients for the Tikhonov filter as a function of *β*. The optimal value was determined to occur at *β* = 125, yielding *C* = 0.7914. Figure B in S1 File confirms that this optimal value holds for the other imaging subjects as well, by showing image reconstructions as a function of *β*.

#### Image domain

**Fig 8:**
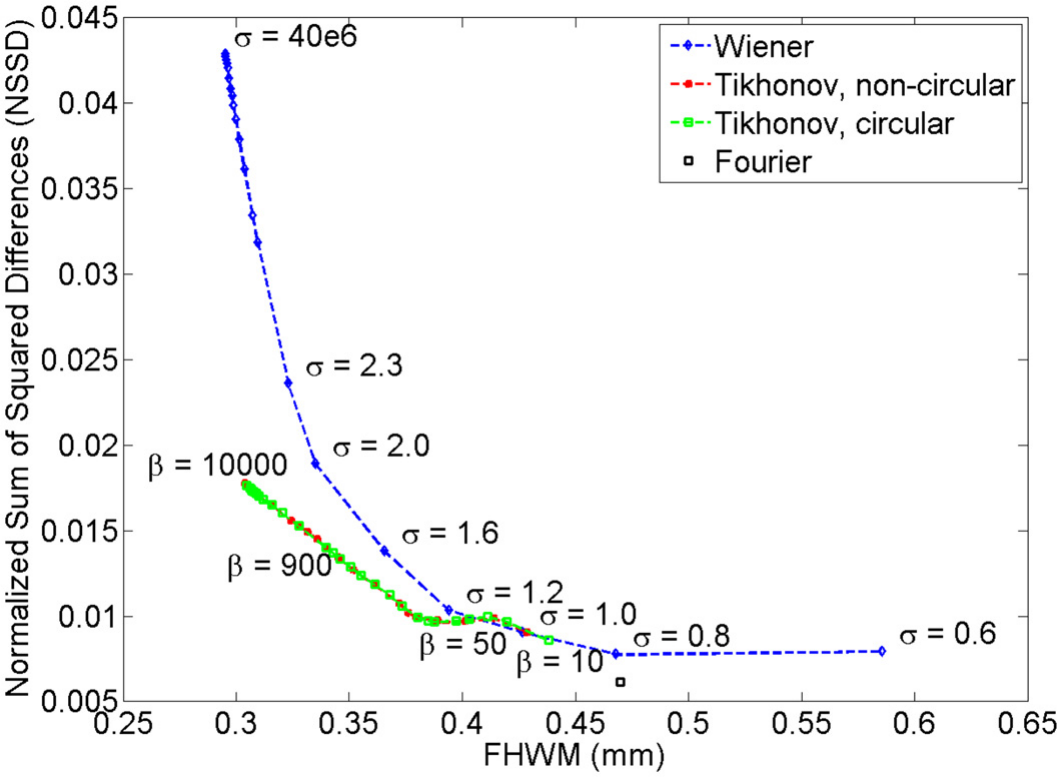
Noise (measured by NSSD) versus resolution (measured by FWHM) curves for various filter choices and parameter settings. The parameter *σ* is expressed in MHz; the parameter *β* is unitless.

Next, we computed noise versus resolution curves by analyzing reconstructions of the pencil point phantom when using the various filters and parameter values. The curves are shown in Fig 8. Note that Fig 8 also shows results for the Tikhonov filter with a circular convolution matrix (i.e. the scanner impulse response *p*_*d*0_(*t*) is assumed to wrap around the edges of the recorded signals). This allows us to assess whether the differences between the Tikhonov (matrix) approach and Fourier/Wiener approaches are mainly due to the different regularization of the deconvolved signals, or simply due to the difference between the non-periodic (non-circular) and periodic (circular) boundary conditions of the matrix and Fourier/Wiener methods. The noise was measured by computing the sum of squared differences between neighboring pixels in a background patch, normalized by the sum of the squares of the intensities in that patch. The background patch was defined as any image region at least 2 mm away from the center of the pencil point. As explained in the Deconvolution filter parameter optimization section, the resolution was measured by fitting 1D Gaussians to line profiles through the reconstructed pencil point, and determining the full width at half maximum (FWHM).

The curves reveal the expected trade-off between noise and resolution: the more smoothing is applied by a deconvolution method, the lower the noise in the reconstructed image, but also the lower the image resolution. The Wiener and Tikhonov methods achieve similar resolutions (FWHMs), but the Tikhonov methods display superior noise suppression. Hence we can consider the Tikhonov methods (with both circular and non-circular convolution models) to outperform the Wiener filter. The Fourier filter produces the poorest FWHM and therefore the blurriest image reconstructions, albeit with the benefit of achieving the lowest image noise levels. In summary, the Tikhonov filter gave the best performance, followed by the Wiener filter and then the Fourier filter. As for the importance of the circular versus non-circular convolution model in the Tikhonov filter, its choice does not appear to substantially impact the filters’ performance. Hence, the difference between the Tikhonov versus Fourier and Wiener filters appears to be the result of the difference in regularization, rather than the difference in their circular versus non-circular convolution models. Finally, note that both the Wiener and Tikhonov curves converge onto a single point as *σ* and *β* increase to infinity, hence limiting their best case FWHM and worst case noise amplification.

#### Parameter optimization summary

The Tikhonov method was found to achieve the most faithful balance of lower and higher frequencies of all three methods, as measured by the highest normalized correlation coefficient of *C* = 0.7914 at *β* = 125 (see Projection domain section). It also achieved a desirable noise versus resolution performance in the image domain (see Fig 8). Following these results, we decided to use *β* = 125 for all Tikhonov image reconstructions shown below. For the Wiener filter, the most faithful balance of lower and higher frequencies was achieved for *σ*’s greater than 100 MHz. Combining the results of the Projection domain section and Fig 8, we decided to use *σ* = 10,000 MHz for all reconstructions shown below. This value yielded a desirable NCC metric of *C* = 0.733 and an image resolution close to the highest observed. This choice does compromise on noise amplification, but this was found not to be problematic in any of our Wiener image reconstructions under standard scanning conditions. In other words, the optimal frequency balance and high image resolution was prioritized over noise suppression. Lastly, the Fourier filter performed poorest in both the projection and image domains.

### Comparison of image reconstructions

In this section, we compare the image quality obtained by each deconvolution method for each of the four imaging subjects, using the optimal parameter values selected above. All imaging subjects were scanned using 180 views and 100 pulses per view. These settings provided a desirable trade-off between (a) reduced streak artifacts (due to higher geometric sampling density) as well as better signal to noise ratios (due to greater signal averaging) and (b) longer scan times, where more views and pulses per view require longer scan times given the fixed rate at which the laser fires its pulses (20 times per second). At 180 views and 100 pulses per view, scan times were approximately 15 minutes. For most of our imaging subjects, increasing the settings beyond 180 views and 100 pulses did not yield noticeable improvements in image quality. The one exception was the mouse brain scan, which was performed with 400 pulses per view. This was done to maintain a high signal to noise ratio despite the fact that the brain’s photoacoustic signal was relatively weak due to the absence of blood.

**Fig 9:**
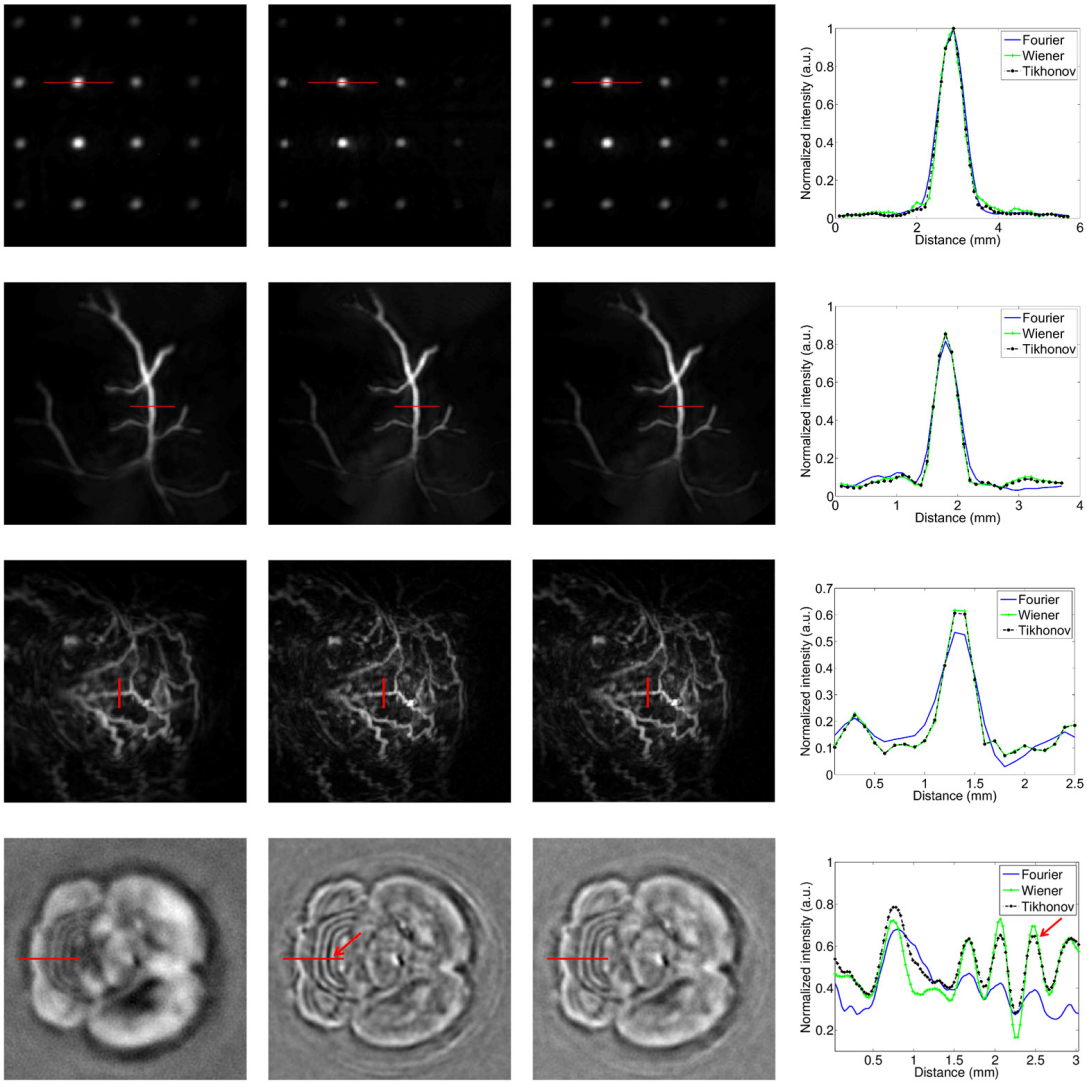
Reconstructions by each deconvolution method. First row: dot phantom. Second row: vessel phantom. Third row: subcutaneous mouse tumor. Fourth row: mouse brain. First column: Fourier filter. Second column: Wiener filter. Third column: Tikhonov method. Fourth column: illustrative line profiles. The image intensities of the reconstructions are normalized (black: 0, white: 1), and the dimensions of the MIP images are 20 × 20 mm.

All reconstructions are shown in Fig 9. The first and second rows show maximum intensity projections (MIP) of the dot and vessel phantoms. The third row shows MIP images of the reconstructed mouse tumor. The highly vascularized nature of the subcutaneous tumor, as well as the tortuous nature of the blood vessels, is clearly discernable. The drop-off in intensity near the edges of the dot phantom, vessel phantom and tumor vasculature is due to the non-uniform illumination by the laser light, and the absence of light fluence compensation in the backprojection algorithm. Next, the fourth row shows a slice through the mouse brain as reconstructed with each deconvolution method. We opted to show slices rather than MIP images for this particular scan because the slices better visualized the fine structures present in the brain. The right-most column of Fig 9 shows intensity profiles through selected features in each of the reconstructions. The locations of the intensity profiles are indicated by red lines in the images. The intensity profiles were used to estimate the full width at half maximum (FWHM) and contrast-to-noise ratio (CNR) of the features. For the brain, we computed the FWM and CNR for the third linear structure from the left that crosses the intensity profile. This structure is indicated by red arrows in the Wiener reconstructed image as well as the brain’s intensity profile plot, and was located approximately 2.5 mm below the surface of the brain.

**Fig 10:**
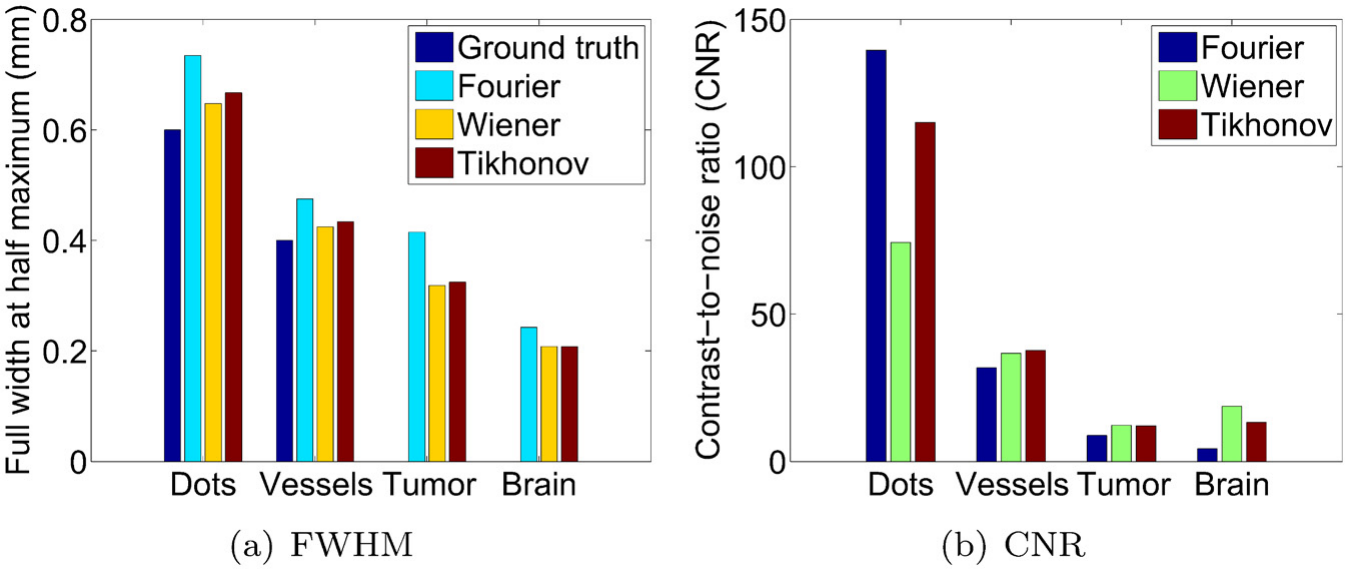
FHWM and CNR of various linear features by the three deconvolution methods. The ground truth FWHM is known only for the artificial phantoms.

Qualitatively, the image reconstructions by the Fourier method appear slightly blurrier than those by the Wiener and Tikhonov methods. This can be appreciated in the images as well as the intensity profiles, and is confirmed by the FWHM estimates in Fig 10(a). The blurrier appearance of the Fourier reconstructions is due to the underrepresentation of low frequencies in the scanner impulse response *p*_*d*0_(*t*) (due to the band-pass nature of the transducer and hence underrepresentation of low frequencies in the transducer impulse response *h*_*t*_(*t*)—see Fig 3). As a result, the Fourier division technique disproportionately boosts lower frequencies in the data space, and consequently in the image space after backprojection. Stated differently, any small and poorly measured magnitudes in the low frequencies of the transducer response end up magnifying the equally poorly measured low frequencies in the recorded signal. As a result, the amplified lower frequencies lead to blurrier image appearances. Note that the higher frequencies in the deconvolved signals tend to be overly amplified as well, due to a similar underrepresentation of higher frequencies in the scanner impulse response *p*_*d*0_(*t*). However, this effect is mitigated by the standard practice of applying a high-frequency suppressing filter (see Methods section) after the Fourier division. While a similar regularization could be applied at lower frequencies, such measures would lead us further away from applying a standard Fourier division deconvolution. Our purpose is to compare the performance of the standard Fourier division deconvolution to alternative methods, namely the Wiener and Tikhonov deconvolution methods. Next, we observe that the difference between the three deconvolution methods is most pronounced for the mouse brain. This is likely due to the fact that the spherical projections of the brain possess more lower frequency content than those of the other imaging subjects. This enables a more obvious demonstration of the difference in the frequency balancing by the various methods. Note in particular that the Tikhonov filter appears to achieve a superior balance between high and low spatial frequencies compared to the Fourier and (admittedly similar) Wiener filters. This observation agrees with the prediction made in the “Tikhonov: optimization of the parameter *β*” section.

For a more quantitative assessment of the reconstructions, we computed the FWHM and CNR of the features shown in the intensity profiles in the right column of Fig 9. Fig 10(a) and 10(b) show the FWHM and CNR values, respectively. For the FWHM results, we observe that the Wiener filter consistently gave the lowest FWHM, which were also closest to the ground truth when known. This indicates that the Wiener filter yielded the highest image resolution, which agrees with our results from the Image domain section and choice of *σ* in the Parameter optimization summary section. It also agrees with the qualitative impression in Fig 9 that the Wiener filter emphasizes the higher frequency content more than the other two methods (in particular, the Wiener filter’s brain reconstruction arguably appears like a high-pass filtered version of the other two reconstructions). The second lowest FWHM values were produced by the Tikhonov filter. This was again to be expected from our choice of *β*, which trades off a faithful balance of frequencies with a slight loss in resolution (see Parameter optimization summary section). The Fourier filter produced the largest estimates, again agreeing with our previous observation that the Fourier filter yields blurrier reconstructions.

Next, the observed trends in the CNR values were not as consistent as those for the FWHM values. The Fourier filter gave the highest CNR for the dot phantom, yet the lowest for the other imaging subjects. However, the Fourier filter’s large CNR value for the dot phantom was caused by a much lower standard deviation of the background, not by a significantly higher contrast (peak-to-trough) value. Since the low standard deviation was due to the blurring effect of the Fourier method, the higher CNR by the Fourier filter did not necessarily correspond to a more desirable image. As for the performance of the other methods on the dot phantom, the Wiener method gave the lowest CNR due to some residual reconstruction artifacts between the dots. Given its high CNR and competitive FWHM, the Tikhonov reconstruction was judged to perform the best for this phantom. For the vessel phantom, the Tikhonov method provided the highest CNR, followed by the Wiener and then Fourier method. For the mouse tumor, the Fourier method’s CNR was again lowest, and the Tikhonov and Wiener method produced a nearly identical CNR. The combination of these results again favors the general use of the Tikhonov filter. Lastly, for the mouse brain reconstructions, the highest CNR was achieved by the Wiener filter, followed by the Tikhonov filter as a close second, and then the Fourier filter. The Wiener filter’s higher CNR was a direct result of a greater feature amplitude in the intensity profile. However, this was due solely to the choice of *σ* in the Wiener filter, favoring higher image resolutions (see Parameter optimization summary section). Hence the Tikhonov filter may still be preferable for its more accurate balancing of lower frequency contrast and higher frequency detail (higher NCC value in the “Tikhonov: optimization of the parameter *β*” section), despite its slightly lower CNR for this particular linear feature.

In summary, while the Wiener method produced slightly sharper images than the Tikhonov method, it did so at the cost of a poorer balance between lower and higher frequency content. In particular, it appeared to underrepresent the lower frequency content of the signals, and by consequence of the reconstructed images. In other words, the Tikhonov filter was deemed to give a better trade-off between image resolution and balancing of low and high frequency content. Note also that the frequency balancing by the Wiener filter could be improved by post-reconstruction low frequency boosting filtering (where frequency filtering is an acceptable and commonly used post-processing technique to improve the interpretability of images). However, we contend that the Tikhonov filter is preferable due to its more faithful frequency balancing *before* any post-processing steps.

### Sensitivity to noise

**Fig 11:**
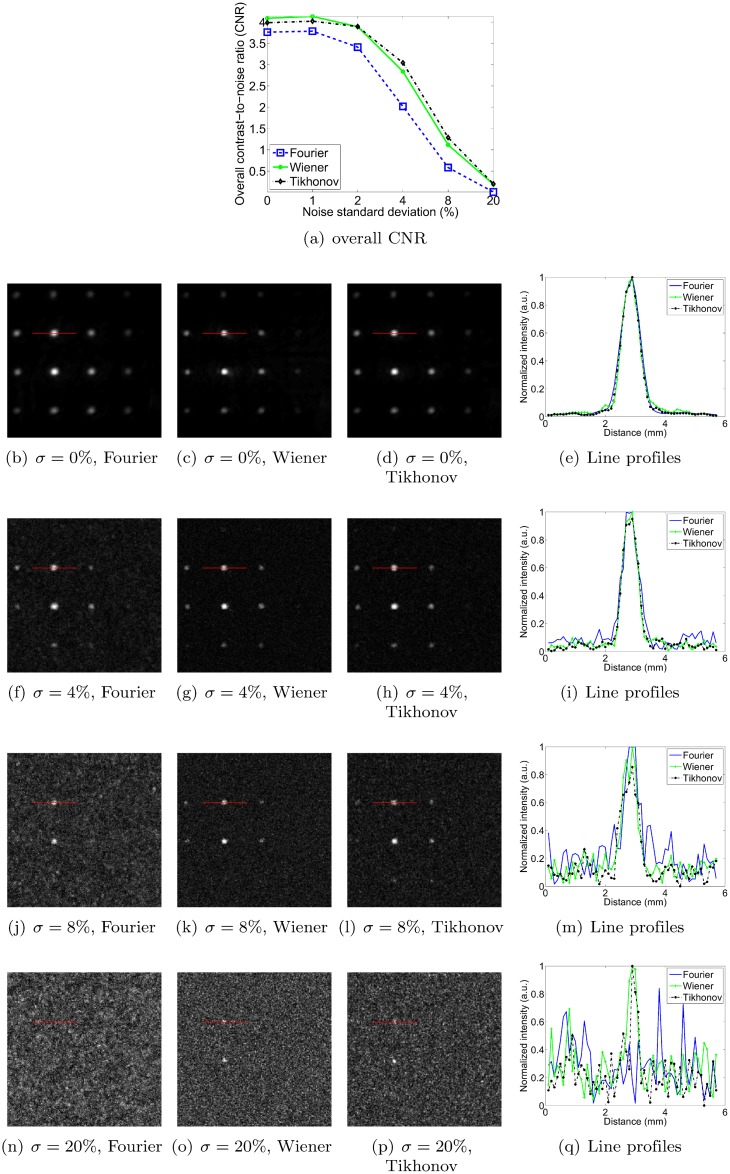
Robustness to noise of each deconvolution filter, as illustrated for the dot phantom. *σ* denotes the standard deviation of the zero-mean Gaussian noise added to the pressure signals, expressed as a percentage of the maximum pressure signal value. Each filter is used at its optimal setting, as determined in the Filter parameter optimization section. The line profiles in the right hand side column have been normalized. The image intensities of the reconstructions are normalized as well (black: 0, white: 1), and the dimensions of the MIP images are 20 × 20 mm.

**Fig 12:**
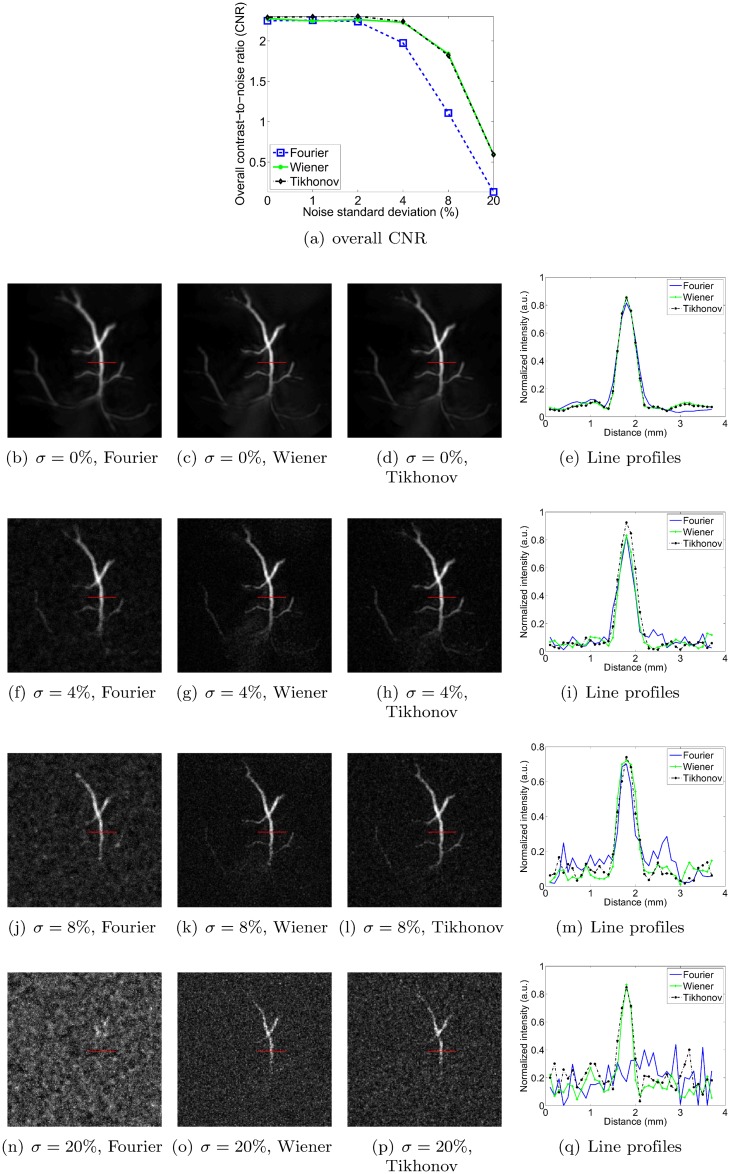
Robustness to noise of each deconvolution filter, as illustrated for the vessel phantom. *σ* denotes the standard deviation of the zero-mean Gaussian noise added to the pressure signals, expressed as a percentage of the maximum pressure signal value. Each filter is used at its optimal setting, as determined in the Filter parameter optimization section. The image intensities of the reconstructions are normalized (black: 0, white: 1), and the dimensions of the MIP images are 20 × 20 mm.

**Fig 13:**
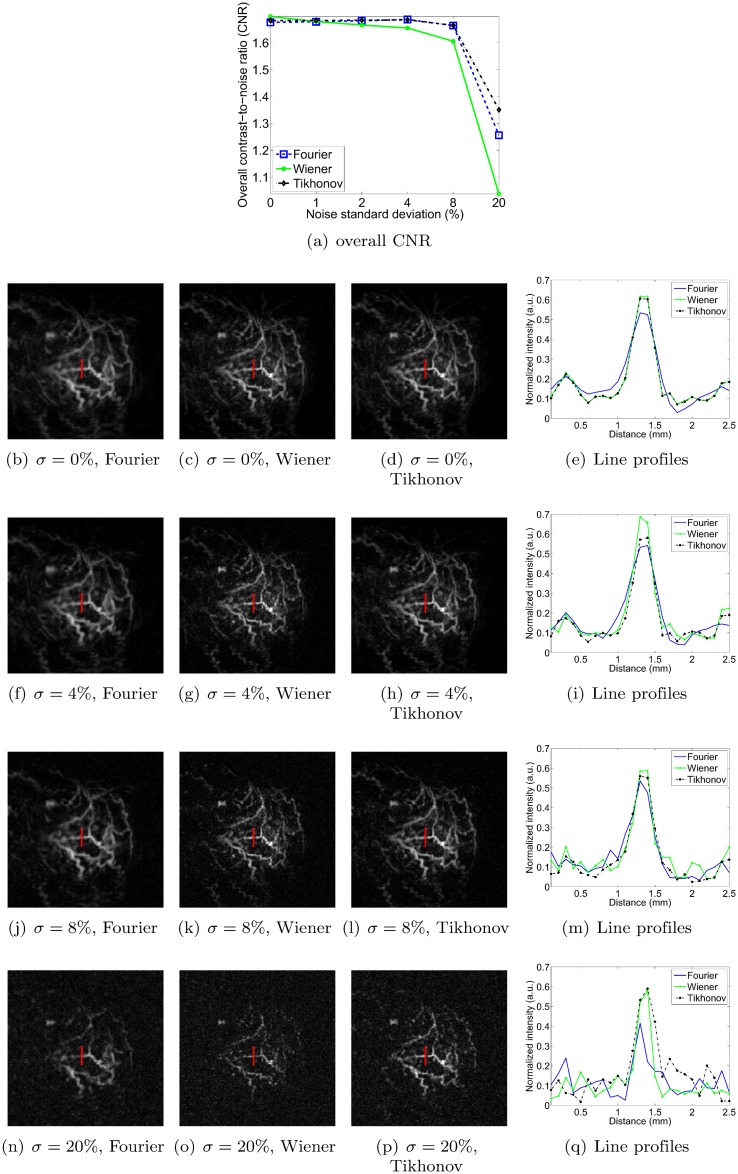
Robustness to noise of each deconvolution filter, as illustrated for the vascularized subcutaneous mouse tumor. *σ* denotes the standard deviation of the zero-mean Gaussian noise added to the pressure signals, expressed as a percentage of the maximum pressure signal value. Each filter is used at its optimal setting, as determined in the Filter parameter optimization section. The image intensities of the reconstructions are normalized (black: 0, white: 1), and the dimensions of the MIP images are 20 × 20 mm.

**Fig 14:**
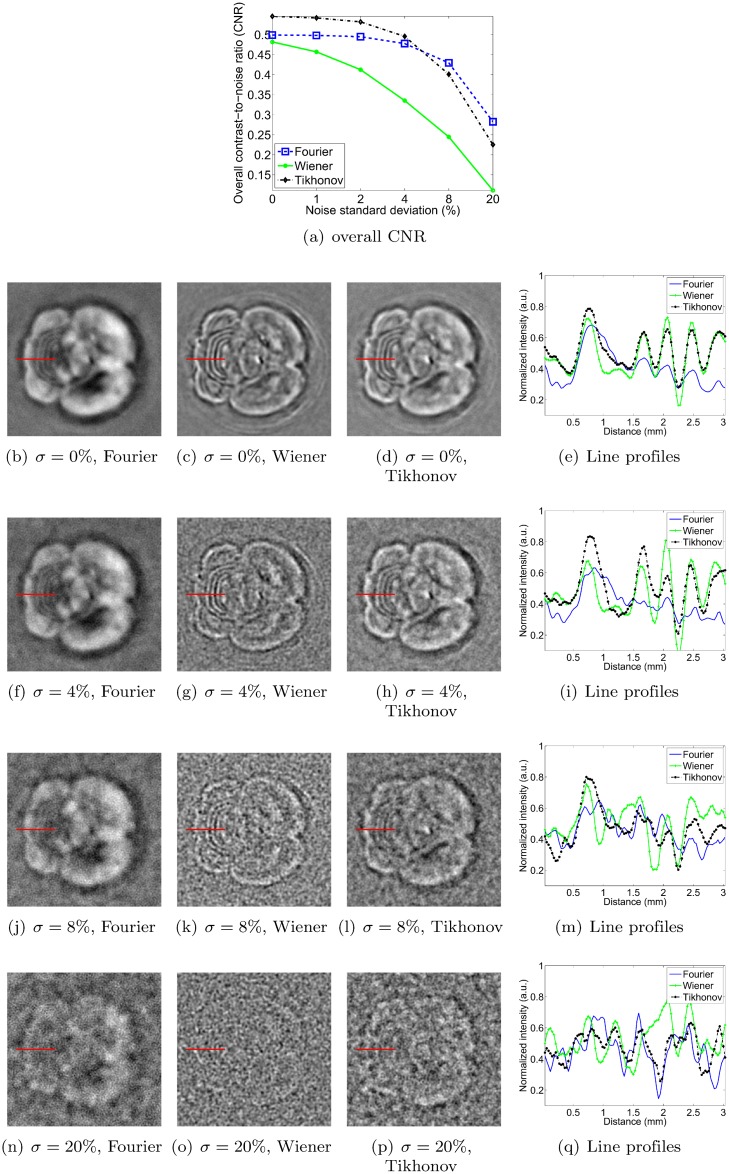
Robustness to noise of each deconvolution filter, as illustrated for the perfused and excised mouse brain. *σ* denotes the standard deviation of the zero-mean Gaussian noise added to the pressure signals, expressed as a percentage of the maximum pressure signal value. Each filter is used at its optimal setting, as determined in the Filter parameter optimization section. The image intensities of the reconstructions are normalized (black: 0, white: 1), and the dimensions of the MIP images are 20 × 20 mm.

In this section, we measure the robustness to noise of each method by adding increasing levels of zero-mean Gaussian noise to the pressure signals. The standard deviation of the noise is expressed as a percentage of the maximum signal amplitude of the original pressure data. Figs 11, 12, 13 and 14 show reconstructions obtained with the three deconvolution methods for different levels of noise. The most obvious effect of the increasing noise levels is to gradually erode the fainter structures in the image; only the brightest features remain visible at the highest noise levels. Figs 11, 12, 13 and 14 also show that the overall CNR of the reconstructions degrades with increasing noise levels. Qualitatively, it can be appreciated that the Tikhonov filter appears to be most robust to the increasing noise levels for all objects. In particular, it visually performs similarly to the Wiener filter for the dot and vessel phantoms, clearly outperforms the Fourier and Wiener filters for the subcutaneous tumor, and arguably performs best for the mouse brain. The performance of the Wiener and Fourier filters, on the other hand, is more variable: the Wiener filter is more robust than the Fourier filter for the dot and vessel phantoms, but the Fourier filter is more robust than the Wiener filter for the subcutaneous tumor and mouse brain scans.

To obtain a more quantitative assessment of performance, we also estimated the overall CNR of the reconstructed images. As explained in the Simulation of noise in scan data section, we opted for a global CNR metric as it proved more robust than one computed from narrow intensity profiles at higher noise levels. Incidentally, note that the line profiles shown in Figs 11, 12, 13 and 14 are shown merely to enable qualitative assessment—they were not used as the basis for the CNR curves shown in panel (a) of each of the respective figures. As expected, the CNR degraded with increasing noise levels for all objects and all methods. For the dot phantom, the CNR by the Wiener filter was marginally higher than that by the Tikhonov filter at lower noise levels. However, the Tikhonov filter proved slightly more robust, showing a slower degradation in CNR as the noise level increased. The Tikhonov method also succeeded in rendering more dots than the Wiener method, as can be seen particularly in the bottom row of dots in the 8% noise reconstruction. The Fourier method clearly performed poorest at all noise levels, both visually and in terms of its CNR curve. For the vessel phantom, the Tikhonov and Wiener methods performed similarly, although the Tikhonov method’s CNR curve showed a slight advantage at lower noise levels. The Fourier method again showed the poorest robustness to the added noise. For the vascularized subcutaneous tumor, the Tikhonov filter was most robust in terms of both overall CNR and the qualitative image appearances, followed by the Fourier method in second place, and the Wiener filter in third.

Lastly, for the mouse brain, the soft mask approach for the overall CNR determination was arguably less meaningful than for the other subjects, since the distinction between foreground and background was less clear. Nonetheless, the trends seen in the CNR curves appear to agree with a qualitative assessment of the reconstructed images. The Tikhonov filter yielded the highest CNR at low noise levels, doing so to a noise level of about 6%, after which the Fourier filter took over. However, the higher CNR of the Fourier reconstructions was the result of noise suppression by excessive blurring. Hence the Tikhonov reconstructions were arguably preferable across the entire range of noise intensities. The Wiener filter clearly displayed the poorest noise amplification of the three methods.

In summary, the Tikhonov L-2 norm filter yielded the highest overall robustness to noise in the recorded pressure signals, as determined by both qualitative assessment of the images and the computed CNR curves.

## Conclusions and Further Work

### Conclusions

In this study, we compared the merits of three methods for deconvolving the pressure data prior to filtered backprojection in photoacoustic tomography. The methods were the classic Fourier division technique, the Wiener filter, and a Tikhonov L-2 norm regularized matrix inversion technique. We compared the final image quality achieved by each deconvolution technique, and evaluated each method’s robustness to noise. Using both qualitative and quantitative assessments, it was found that the Tikhonov filter achieved the most accurate balance of frequencies, a competitive image resolution and contrast-to-noise ratio, as well as the greatest robustness to noise. While the Wiener filter achieved a similar resolution, it tended to underrepresent the lower frequency content of the deconvolved signals. It was also less robust to noise in the recorded pressure signals. The performance of the Fourier filter was deemed to be the poorest, based on the reconstructed images’ lowest resolution (blurriest appearance), generally lowest contrast-to-noise ratio, as well as lowest robustness to noise. These trends were observed across imaging subjects of various appearances. Overall, the Tikhonov filter was deemed to produce the most desirable image reconstructions. Lastly, the results presented in this work demonstrate that the final photoacoustic image quality is highly dependent on the particular method used for signal deconvolution, as well as its proper optimization.

### Further work

In future work, we aim to examine whether post-reconstruction boosting of lower frequency content could optimize the lower frequency content versus image resolution trade-off of the Wiener deconvolution filter. We also aim to investigate additional deconvolution methods such as Kalman filters, L-1 norm regularized deconvolution, and non-negativity constrained matrix deconvolution. We also note that the deconvolution operation is an integral part of reconstruction algorithms other than filtered backprojection as well, for example iterative reconstruction methods. Hence, we aim to examine the effect of different deconvolution schemes (such as the Fourier, Tikhonov, Wiener, and Kalman filters) on the performance of such alternative reconstruction algorithms as well.

## Supporting Information

S1 File Filter parameter optimization. **Figure A, Image reconstructions with Wiener deconvolution for different values of *σ***. Note that the brain slice appears shrunken without signal deconvolution. This is because the deconvolution also corrects for a slight time delay between the true arrival of the pressure waves at the detector surface and the actual pressure signal recorded by the scanner. This delay was present in all scans equally, including the scan in which the scanner impulse response function *p*_*d*0_(*t*) was measured. Without the deconvolution’s correction for this time delay, the backprojected signals are slightly shifted and misaligned in space. This misalignment in turn results in the shrunken appearance of the reconstructed brain slice. The spatial location of the brain slice within the reconstructed volume is identical for all reconstructions shown. The image intensities of the reconstructions are normalized (black: 0, white: 1), and the dimensions of the MIP images are 20 × 20 mm. **Figure B, Image reconstructions with Tikhonov deconvolution for different values of *β***. Note that the brain slice appears shrunken without signal deconvolution. This is because the deconvolution also corrects for a slight time delay between the true arrival of the pressure waves at the detector surface and the actual pressure signal recorded by the scanner. This delay was present in all scans equally, including the scan in which the scanner impulse response function *p*_*d*0_(*t*) was measured. Without the deconvolution’s correction for this time delay, the backprojected signals are slightly shifted and misaligned in space. This misalignment in turn results in the shrunken appearance of the reconstructed brain slice. The spatial location of the brain slice within the reconstructed volume is identical for all reconstructions shown. The image intensities of the reconstructions are normalized (black: 0, white: 1), and the dimensions of the MIP images are 20 × 20 mm.
(PDF)
