# Supplementary material for: Comparison of Deconvolution Filters for Photoacoustic Tomography
Source: PLoS One. 2016 Mar 31;11(3):e0152597. doi: 10.1371/journal.pone.0152597 (PMC4816281; doi:10.1371/journal.pone.0152597)
Supplement: S1 File — Figure A, Image reconstructions with Wiener deconvolution for different values of σ. Note that the brain slice appears shrunken without signal deconvolution. This is because the deconvolution also corrects for a slight time delay between the true arrival of the pressure waves at the detector surface and the actual pressure signal recorded by the scanner. This delay was present in all scans equally, including the scan in which the scanner impulse response function pd0(t) was measured. Without the deconvolution’s correction for this time delay, the backprojected signals are slightly shifted and misaligned in space. This misalignment in turn results in the shrunken appearance of the reconstructed brain slice. The spatial location of the brain slice within the reconstructed volume is identical for all reconstructions shown. The image intensities of the reconstructions are normalized (black: 0, white: 1), and the dimensions of the MIP images are 20 × 20 mm. Figure B, Image reconstructions with Tikhonov deconvolution for different values of β. Note that the brain slice appears shrunken without signal deconvolution. This is because the deconvolution also corrects for a slight time delay between the true arrival of the pressure waves at the detector surface and the actual pressure signal recorded by the scanner. This delay was present in all scans equally, including the scan in which the scanner impulse response function pd0(t) was measured. Without the deconvolution’s correction for this time delay, the backprojected signals are slightly shifted and misaligned in space. This misalignment in turn results in the shrunken appearance of the reconstructed brain slice. The spatial location of the brain slice within the reconstructed volume is identical for all reconstructions shown. The image intensities of the reconstructions are normalized (black: 0, white: 1), and the dimensions of the MIP images are 20 × 20 mm. (PDF) [file pone.0152597.s001.pdf]

S1 File: Filter parameter optimization

Wiener filter

Figure A shows image reconstructions of all subjects with the Wiener filter for illustrative values of σ. The reconstructions illustrate that the best values of σ as predicted by the impulse signal deconvolution experiment (see the Filter parameter optimization section in the main manuscript) also give desirable image reconstructions when applied to other data sets. The top row of Figure A shows reconstructions by filtered backprojection without prior signal deconvolution for the sake of comparison.

Tikhonov filter

Figure B shows image reconstructions of all subjects with the Tikhonov filter for illustrative values of β. Again, the results illustrate that the optimal value of β as determined by the impulse signal deconvolution experiment generates desirable images for other imaging subjects as well. For the mouse brain in particular, it can be appreciated that $\beta = 125$ achieves a better trade-off between low frequency contrast and higher frequency detail than either $\beta = 10$ or $\beta = 500$. The difference is more subtle in the case of the blood vessels in the subcutaneous mouse tumor model. However, in this case one can still observe that $\beta = 10$ appears more blurred than the other reconstructions. Upon a closer look, one can also see that $\beta = 125$ yields a slightly higher contrast for some of the structures compared to $\beta = 500$ (see red arrows). In addition, the $\beta = 500$ reconstruction appears slightly grainier.

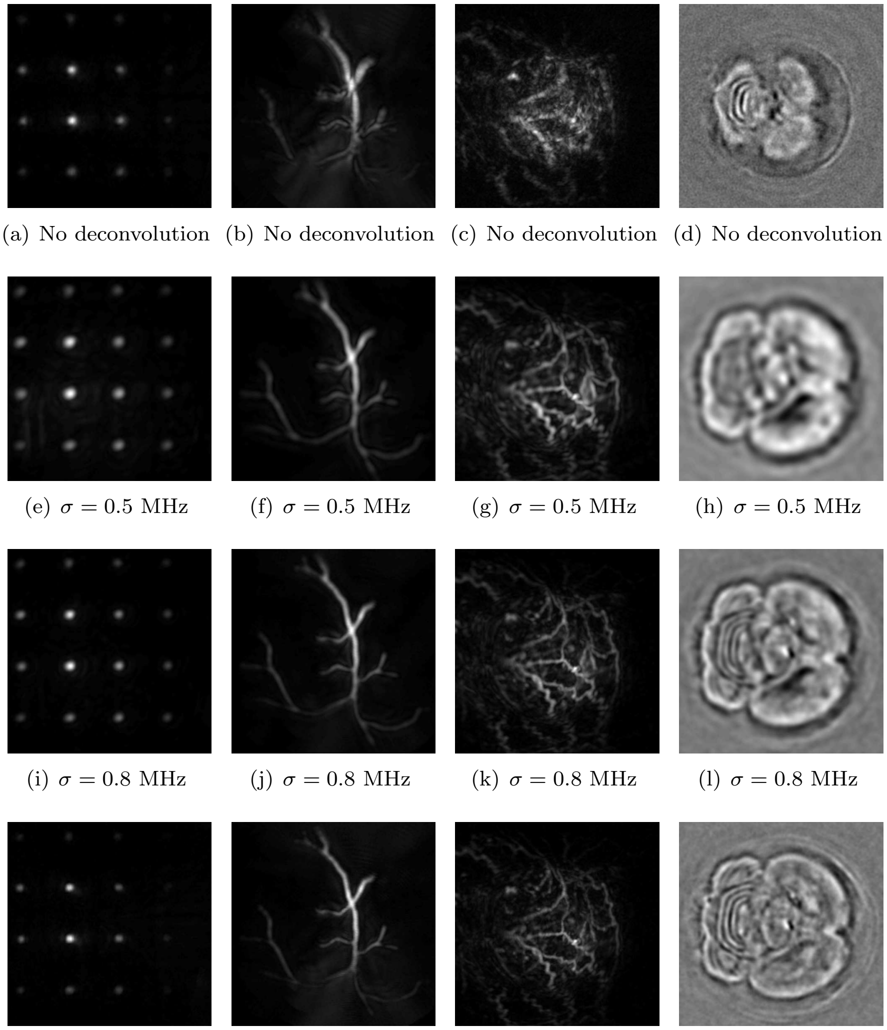

(a) No deconvolution (b) No deconvolution (c) No deconvolution (d) No deconvolution

(e) $\sigma = 0.5$ MHz (f) $\sigma = 0.5$ MHz (g) $\sigma = 0.5$ MHz (h) $\sigma = 0.5$ MHz

(i) $\sigma = 0.8$ MHz (j) $\sigma = 0.8$ MHz (k) $\sigma = 0.8$ MHz (l) $\sigma = 0.8$ MHz

(m) $\sigma = 10,000$ MHz (n) $\sigma = 10,000$ MHz (o) $\sigma = 10,000$ MHz (p) $\sigma = 10,000$ MHz

Figure A: Image reconstructions with Wiener deconvolution for different values of σ. Note that the brain slice appears shrunken without signal deconvolution. This is because the deconvolution also corrects for a slight time delay between the true arrival of the pressure waves at the detector surface and the actual pressure signal recorded by the scanner. This delay was present in all scans equally, including the scan in which the scanner impulse response function $p_{d0}(t)$ was measured. Without the deconvolution's correction for this time delay, the back-projected signals are slightly shifted and misaligned in space. This misalignment in turn results in the shrunken appearance of the reconstructed brain slice. The spatial location of the brain slice within the reconstructed volume is identical for all reconstructions shown. The image intensities of the reconstructions are normalized (black: 0, white: 1), and the dimensions of the MIP images are 20 x 20 mm.

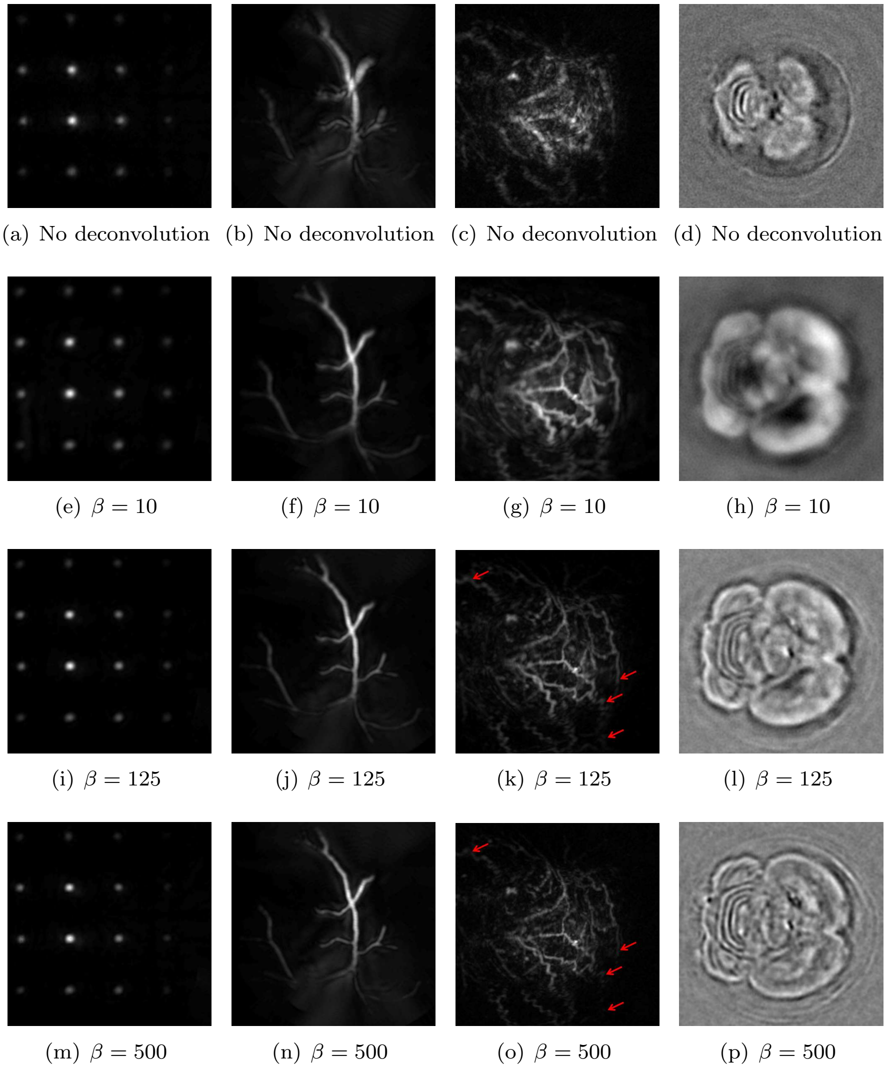

Figure B: Image reconstructions with Tikhonov deconvolution for different values of β. Note that the brain slice appears shrunken without signal deconvolution. This is because the deconvolution also corrects for a slight time delay between the true arrival of the pressure waves at the detector surface and the actual pressure signal recorded by the scanner. This delay was present in all scans equally, including the scan in which the scanner impulse response function $p_{d0}(t)$ was measured. Without the deconvolution's correction for this time delay, the backprojected signals are slightly shifted and misaligned in space. This misalignment in turn results in the shrunken appearance of the reconstructed brain slice. The spatial location of the brain slice within the reconstructed volume is identical for all reconstructions shown. The image intensities of the reconstructions are normalized (black: 0, white: 1), and the dimensions of the MIP images are 20 x 20 mm.
